# Bleomycin Induces Molecular Changes Directly Relevant to Idiopathic Pulmonary Fibrosis: A Model for “Active” Disease

**DOI:** 10.1371/journal.pone.0059348

**Published:** 2013-04-02

**Authors:** Ruoqi Peng, Sriram Sridhar, Gaurav Tyagi, Jonathan E. Phillips, Rosario Garrido, Paul Harris, Lisa Burns, Lorena Renteria, John Woods, Leena Chen, John Allard, Palanikumar Ravindran, Hans Bitter, Zhenmin Liang, Cory M. Hogaboam, Chris Kitson, David C. Budd, Jay S. Fine, Carla MT. Bauer, Christopher S. Stevenson

**Affiliations:** 1 DTA Inflammation, Hoffmann-La Roche Inc., pRED, Pharma Research and Early Development, Nutley, New Jersey, United States of America; 2 Translational Research Sciences, Hoffmann-La Roche Inc., pRED, Pharma Research and Early Development, Nutley, New Jersey, United States of America; 3 Non-Clinical Safety, Hoffmann-La Roche Inc., pRED, Pharma Research and Early Development, Nutley, New Jersey, United States of America; 4 Department of Pathology, University of Michigan Medical School, Ann Arbor, Michigan, United States of America; 5 Imperial College London, National Heart and Lung Institute, Centre for Respiratory Infections, Respiratory Pharmacology Group, Pharmacology and Toxicology Section, London, United Kingdom; Comprehensive Pneumology Center, Germany

## Abstract

The preclinical model of bleomycin-induced lung fibrosis, used to investigate mechanisms related to idiopathic pulmonary fibrosis (IPF), has incorrectly predicted efficacy for several candidate compounds suggesting that it may be of limited value. As an attempt to improve the predictive nature of this model, integrative bioinformatic approaches were used to compare molecular alterations in the lungs of bleomycin-treated mice and patients with IPF. Using gene set enrichment analysis we show for the first time that genes differentially expressed during the fibrotic phase of the single challenge bleomycin model were significantly enriched in the expression profiles of IPF patients. The genes that contributed most to the enrichment were largely involved in mitosis, growth factor, and matrix signaling. Interestingly, these same mitotic processes were increased in the expression profiles of fibroblasts isolated from rapidly progressing, but not slowly progressing, IPF patients relative to control subjects. The data also indicated that TGFβ was not the sole mediator responsible for the changes observed in this model since the ALK-5 inhibitor SB525334 effectively attenuated some but not all of the fibrosis associated with this model. Although some would suggest that repetitive bleomycin injuries may more effectively model IPF-like changes, our data do not support this conclusion. Together, these data highlight that a single bleomycin instillation effectively replicates several of the specific pathogenic molecular changes associated with IPF, and may be best used as a model for patients with active disease.

## Introduction

Idiopathic pulmonary fibrosis (IPF) is a devastating disease characterized by excessive matrix deposition that disrupts the normal architecture of the lung parenchyma. The key pathological features of IPF include fibroblastic foci that are highly synthetic, areas of epithelial cysts associated with the honeycombing appearance of the lung, and mild lymphoplasmacytic interstitial inflammation that is associated with areas of type II cell hyperplasia [1]. Unfortunately current therapies have not substantially impacted disease progression and most patients succumb to respiratory failure with a median survival of approximately 2 to 4 years after diagnosis [2].

The lack of effective therapies is arguably due to an incomplete understanding of the molecular mechanisms driving the disease and the failure of preclinical experimental models to correctly predict the clinical efficacy of several molecules [3]. The bleomycin model is the most commonly used *in vivo* system for investigating candidate therapies. Its failure as a prognostic tool may be ascribed to the fact that the model has not been well characterized in terms of identifying the clinically relevant molecular changes and when they occur. Bleomycin is well understood to induce lung injury that results in an acute inflammatory response that is unlikely to reflect the processes driving the disease in patients. The inflammatory phase is, however, followed by fibrotic changes that replicate certain pathological features consistent with those associated with IPF. Therefore, some have argued that it may be more appropriate to evaluate compounds after or during the onset of the fibrosis phase of the response (i.e. using therapeutic dosing regimens), which may be a more disease-relevant paradigm [3]. Unfortunately, whether there is consistency between the molecular changes that occur during the fibrosis phase of the model and IPF has never been directly assessed. For instance, while tumor growth factor β (TGFβ) is clearly a driver of the remodeling process in the bleomycin model [4], its contribution to disease progression in IPF is currently unknown. Additionally, given the heterogeneity of the disease amongst patients (including its rate of progression), investigating whether the bleomycin model accurately reflects disease mechanisms for all IPF patients or specific subsets will be important for translating findings from the model to the appropriate patient population. Finally, a more recent approach to improve the bleomycin model has been to use repetitive bleomycin challenges, which has been argued to more accurately reflect the temporal and spatial heterogeneity of the pathological changes associated with the disease [5], [6]; however, whether this modification to the system offers significant advantages over the traditional one-hit model remains unclear.

Using classic histopathology and physiology methods, we report that the repetitive model offered no significant improvement over the single challenge model. Integrative bioinformatic and pharmacological approaches revealed corresponding molecular changes in the lungs of bleomycin-treated mice and IPF patients, especially in genes associated with mitosis and extracellular matrix signaling. Interestingly, these same pathways appeared to be altered in fibroblasts isolated from IPF patients with rapidly progressing, but not slowly progressing disease. It did not appear that these changes in expression were directly associated with TGFβ signaling and furthermore, an inhibitor to the TGFβR1 (activin-like kinase 5, ALK-5) could not completely attenuate bleomycin-induced fibrosis in mice. These data support the premise that the bleomycin model can recapitulate many of the complex profibrotic responses that are also elevated in the lungs of IPF patients, particularly in patients with active disease.

## Results

### Inflammatory Changes after a Single or Repeated Bleomycin Challenge

**Figure 1:**
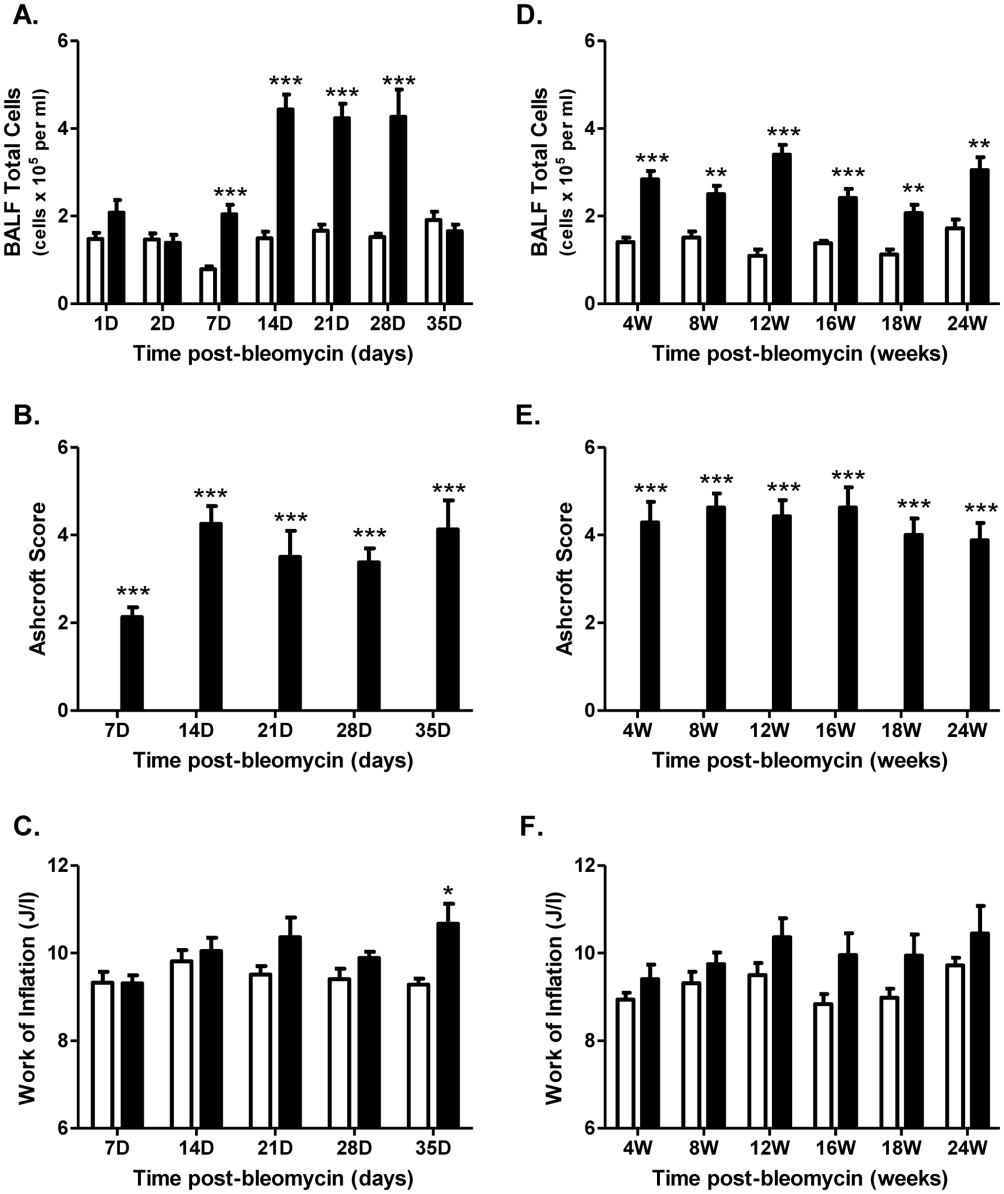
Bleomycin induces inflammatory and fibrotic changes in the lungs of mice. The numbers of BALF inflammatory cells increased after either a single (**A**) or repetitive (**D** bleomycin (black bars) instillation compared to the saline treated controls (white bars). Both a single (**B**) and repetitive (**E**) instillation of bleomycin led to increased fibrotic tissue in the lung. Changes in work of inflation (WoI), a measure of lung mechanics, were minimal in both the single (**C**) and repetitive (**F**) systems. Data are expressed as mean ± SEM of n = 7–8 mice. Significance (relative to the time-matched control at each time point) was determined using a Student’s t-test and is denoted as follows: *p<0.05; **p<0.01; and ***p<0.001.

To establish a dose of bleomycin that induced fibrosis but did not result in mortality, a preliminary bleomycin dose-response study was performed. Bleomycin induced a dose-dependent increase in lung fibrosis (Figure S1). Significant mortality was observed in the groups of mice dosed with either 3 U/kg (19%) or 5 U/kg (50%). Given that a 2 U/kg dose did not cause mortality and induced a submaximal fibrotic response that resulted in lung function changes (data not shown), all subsequent studies were performed using this dose. A single topical challenge with bleomycin induced an acute inflammatory response (Figures 1A) consisting primarily of neutrophils recovered in the bronchoalveolar lavage fluid (BALF) (Figure S2A). The acute response rapidly diminished with time and was supplanted with increasing numbers of lymphocytes and macrophages in the airways (Figures S2B and S2C, respectively). After 35 days, the numbers of cells in the BALF of mice treated with bleomycin returned to the levels observed from saline treated controls with the exception of lymphocytes, which were still slightly elevated.

In response to repetitive bleomycin administration, we observed increased inflammatory cell infiltrate in the BALF of mice (Figure 1D). Interestingly, while BALF neutrophils were initially at low levels (similar to what was observed 4 weeks after a single bleomycin challenge) numbers increased again 12 weeks after the first exposure and remained at elevated levels for the duration of the study (Figure S2D). The levels of lymphocytes (Figure S2E) and macrophages (Figure S2F) in the BALF remained increased throughout the study, although lymphocytes numbers did begin to diminish slightly over time.

### Lung Fibrosis and Lung Function Changes Induced by Bleomycin Administration

A single bleomycin challenge led to an increase in lung fibrosis scores 7 days after administration before plateauing from 14 days onwards (Figure 1B). Interestingly collagen staining in lung sections (Figure S3A) and BALF levels of hydroxyproline (HP) (Figure S3B) were at maximal levels 7 days post-bleomycin instillation. While HP levels resolved over time, lung fibrosis scores and collagen levels in the lung sections remained relatively stable. Changes in lung mechanics, specifically *H* (lung tissue elastance) and work of inflation (WoI) correlated significantly to collagen levels in the lung (Figures S4A and S4B), although the changes were relatively modest and significant at 35 days post-administration (Figure 1C). The areas of fibrosis that stained positive for collagen I (Figure S5D) were proximal to regions positive for fibroblast activation markers, such as α-smooth muscle actin (Figure S5E), as well as a collagen-specific molecular chaperone, heat-shock protein 47 (HSP47) (Figure S5F).

Repetitive administration of bleomycin also led to moderate lung fibrosis (Figure 1E) with approximately 3% of the lung staining positive for collagen I (Figure S3C). Similar to the single challenge model, fibrosis was still present several weeks after bleomycin administration. Interestingly, BALF levels of HP increased up to 8 weeks after the first challenge, but subsequently declined with time returning to near baseline levels at the 18 and 24 week time points (Figure S3D). Analogous to the single challenge model, changes in lung mechanics were modest (Figure 1F) and correlated positively with collagen staining (Figures S4C and S4D).

While changes in lung mechanics were measureable, the magnitude was limited possibly due to the poor sensitivity of pulmonary function tests in small animals. As an alternative to measuring lung mechanics we longitudinally assessed the exercise capacity of mice treated with bleomycin. In a separate study, bleomycin-treated mice clearly had reduced exercise capacity during the fibrotic phase of the response (Figure S6), suggesting that exercise capacity may be a more robust alternative to examinations of lung mechanics.

### Histopathological Features of the Single and Repetitive Bleomycin Challenge Models

**Figure 2:**
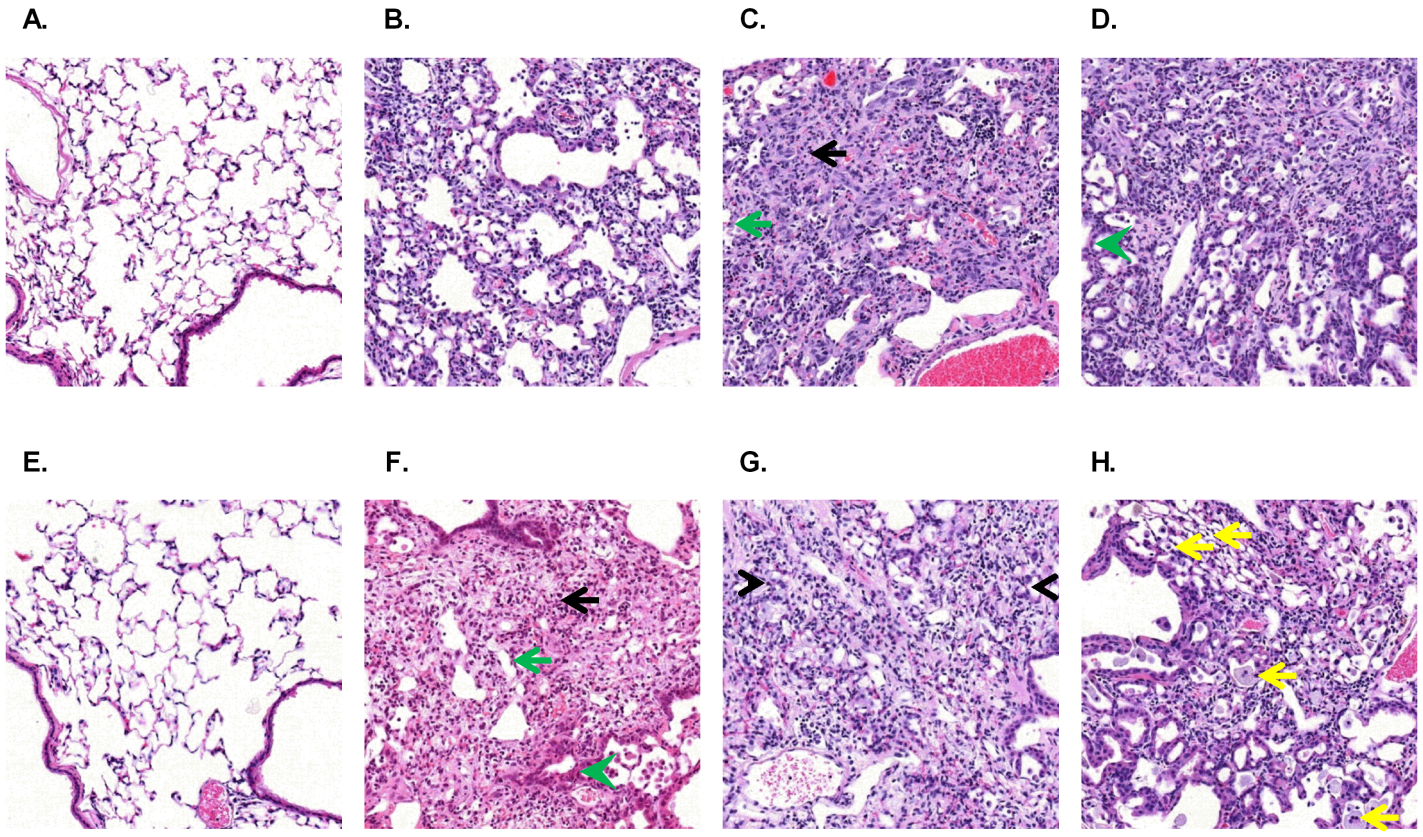
Bleomycin induces inflammation, fibrosis, and epithelial remodeling. Representative images are shown from saline treated controls (**A**), as well as 7 days (**B**), 21 days (**C**), and 35 days (**D**) after a single bleomycin instillation. Additionally, representative images are shown from saline treated controls (**E**), as well as 2 months (**F**), 4 months (**G**), and 6 months (**H**) after the start of the repetitive bleomycin instillations. Areas of alveolar (black arrows) and interstitial (green arrows) fibrosis were clearly present in both models as were areas of bronchoalveolar hyperplasia (green arrow heads). There were also areas of partially collapsed (**D**) and thickened alveoli (**G**) that were often lined by cuboidal bronchoalveolar epithelial cells (black arrow heads) in both systems. At the latest time point examined (**H**), there were also partially collapsed septa, macrophages, and multinucleate giant cells observed in the alveolar space (yellow arrows). Images were captured at 200X magnification of H&E stained lung tissue sections.

During the first week following a single bleomycin instillation the response was primarily characterized by lung inflammation. Inflammatory cells in the alveolar interstitium and alveolar spaces, and proteinaceous fluid were consistently observed with few fibroblasts present (Figure 2B). During the following two weeks, alveolar and interstitial fibrosis became progressively more prominent with partial to complete effacement of the alveoli (Figure 2C). By 5 weeks post-bleomycin administration, the changes were more heterogeneous with fibrosis interspersed with small, partially collapsed alveoli and alveoli lined by bronchoalveolar epithelial cells (Figure 2D).

In the repetitive bleomycin model, each time point presented similar histological features observed in the single challenge model (Figure 2F) – i.e. inflammation, alveolar and interstitial fibrosis with obliteration of alveolar spaces, and bronchoalveolar hyperplasia. With chronicity, the fibrosis became progressively interstitial with thickened alveolar septa becoming more well-defined (Figure 2G). These thickened alveoli were often lined by cuboidal bronchoalveolar epithelial cells that were rarely ciliated. At later time points the fibrosis was mainly interstitial in pattern (Figure 2H). The alveolar spaces were often filled with finely granular to homogenous amphophilic material that was often phagocytized by infiltrating macrophages and multinucleate giant cells, and neutrophils were occasionally observed. Partially collapsed septa (lined by Type I pneumocytes) were also often observed at later time points. In summary, the histopathological changes observed after either a single or repetitive bleomycin instillation were very similar.

### Temporal Gene Expression Responses to Bleomycin

**Figure 3:**
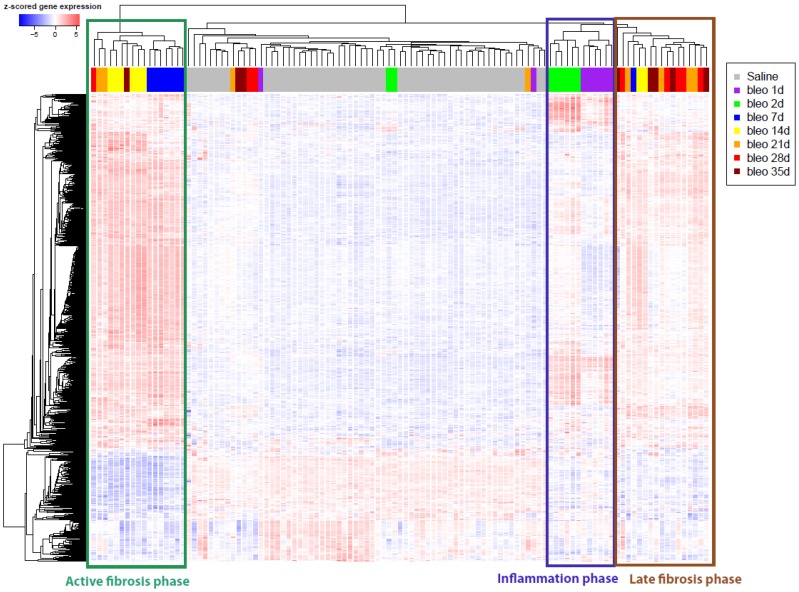
Unsupervised hierarchical clustering of genes differentially expressed between bleomycin and saline treated mice across time points. A union of 730 genes differentially expressed between bleomycin and saline treatments (fold-difference >2, false discovery rate (FDR) <0.05) were clustered across all mouse samples. Three distinct clusters of genes were revealed corresponding to phases of the bleomycin response, with most up-regulated genes being specific to the 7–14 days post-bleomycin treatment clusters. Inflammation phase (1–2 days) samples also clustered together and showed up-regulation of a subset of genes, while late fibrosis phase (21–35 days) samples also showed moderate up-regulation of a subset of genes.

Given that repetitive bleomycin challenges did not accentuate the fibrotic changes observed, all subsequent studies focused on examining changes after a single bleomycin challenge. Microarray analysis revealed 3 distinct temporal phases of the response to bleomycin treatment. Unsupervised hierarchical clustering of the 730 genes commonly altered across time points in response to bleomycin revealed an inflammation phase (days 1–2), an active fibrosis phase (days 7–14) and a late fibrosis phase (days 21–35), with saline-treated samples clustering separately (Figure 3). Peak gene expression responses to bleomycin occurred between days 7 and 14 post-treatment, consistent with the observed fibrotic responses measured in BALF (HP) and lung tissue (Ashcroft scores) (refer to Figures S3B and 1B, respectively). Although there were far fewer differentially expressed genes in the later time points, several genes remained altered 35 days after bleomycin treatment, suggesting that any resolution in gene expression was incomplete at the end of the study.

Genes altered during the inflammation phase generally consisted of immune response and wound healing genes including various cytokines and chemokines. In addition to genes associated with immune and wound healing categories, genes altered during the active fibrosis phase were also associated with cell cycle-related processes. These changes in gene expression were validated by the increased expression of inflammatory cytokines early in the response to bleomycin (Figures S7A–D), followed by increases in pro-fibrotic mediators such as TGFβ and lysophosphatidic acid (LPA) (Figures S7E–F) that coincided with the increased matrix deposition in the lung. Cell cycle genes generally reverted back to control levels 21 days after bleomycin treatment, while several immune and wound healing genes remained up-regulated.

### Enrichment of Fibrosis-related Signaling Pathway in Response to Bleomycin

**Figure 4:**
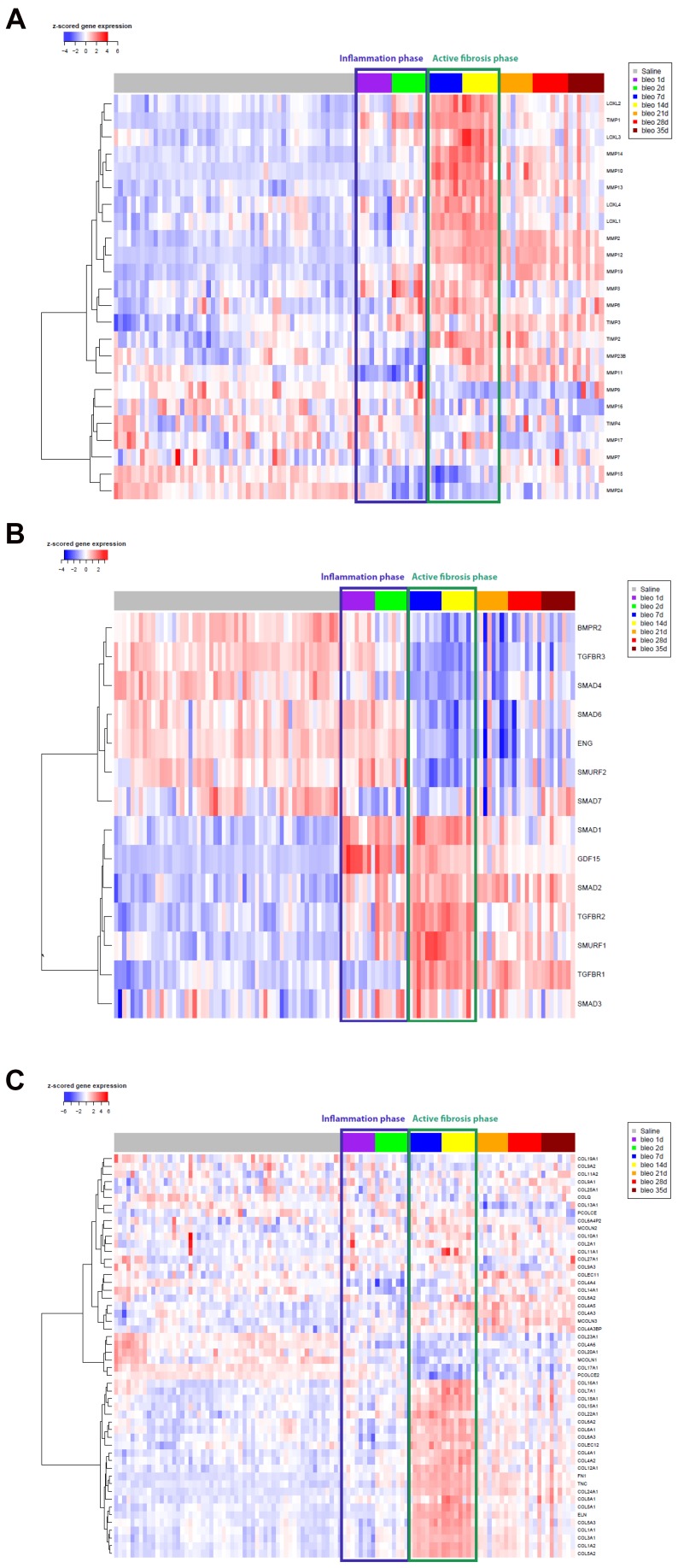
Supervised hierarchical clustering of custom panels of genes across all mouse samples. Clustering of genes was performed for a panel of matrix metalloproteases (MMPs) and lysyl oxidase-like (LOXL) enzymes (**A**), of regulators of TGFβ signaling (B), and genes encoding matrix proteins (**C**) where samples were ordered based on treatment and time point.

Clustering analysis of genes involved in fibrosis-related pathways revealed preferential up-regulation of genes during the active fibrosis phase. Matrix metalloprotease (MMP) genes including MMP12, MMP19, as well as lysyl oxidase-like (LOXL) genes implicated in the pathogenesis of fibrotic diseases were induced from 7 days onwards following bleomycin treatment (Figure 4A). Positive regulators of TGFβ signaling including TGFBR1, TGFBR2, and SMADs 1–3 were up-regulated during the fibrosis phases of the bleomycin response, whereas negative regulators such as inhibitory SMADs (e.g. SMAD6 and SMAD7) were down-regulated (Figure 4B). A large set of collagen genes were also up-regulated during the active fibrosis phase and several remained increased during the late fibrosis phase (Figure 4C). Genes from other signaling pathways implicated in IPF, including Wnt signaling genes and genes altered in response to phosphatidylinositol 3-kinase (PI3K) activity (Figures S8A and S8B, respectively), were also induced during the active fibrosis phase and several of the Wnt signaling genes remained elevated during the late fibrosis phase. A set of genes associated with alternative macrophage activation was also shown to be induced during the active fibrosis phase (Figure S8C), suggesting a role for these cells in the pathogenesis of pulmonary fibrosis.

### Bleomycin Response Signatures are Altered in Clinical IPF Datasets

**Figure 5:**
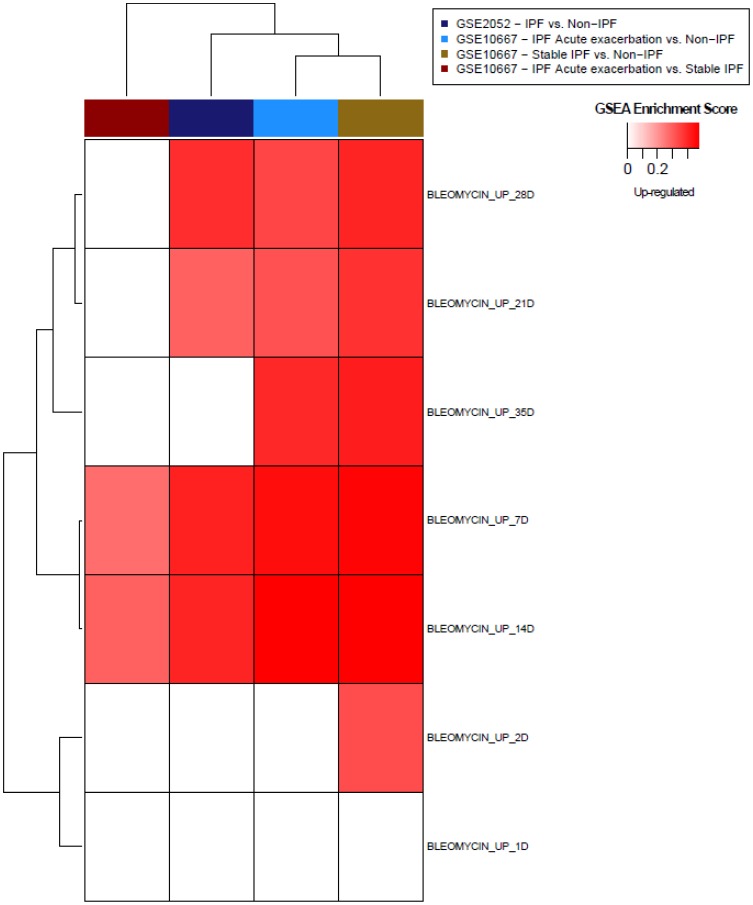
Heatmap of gene set enrichment of mouse bleomycin-induced signatures in clinical IPF datasets. GSEA was performed for gene sets comprised of up-regulated genes in response to bleomycin at each time point from the mouse model. Enrichment of each gene set (denoted in rows) was determined against ranked lists of genes from clinical datasets comparing IPF vs. non-IPF conditions as well as IPF with acute exacerbation vs. stable IPF from two datasets (GSE2052, GSE10667, denoted in columns). Enrichment scores were plotted in a heatmap where gene sets enriched in IPF samples (nominal p<0.05, FDR <0.25) were denoted in red with intensity based on enrichment score (calculated in GSEA).

The translational relevance of the bleomycin response signatures were assessed in clinical IPF datasets using gene set enrichment analysis (GSEA). Gene expression data from two publicly available clinical IPF datasets were compiled: GSE2052 [7], GSE10667 [8]. GSE2052 assayed lung tissue from patients with IPF and non-IPF control subjects. GSE10667 sampled expression from lung tissue of patients with IPF, patients with IPF who suffered an exacerbation, and non-IPF control subjects. Using up-regulated bleomycin response genes across each time point as separate gene sets, GSEA revealed a significant enrichment of the active fibrosis phase signatures in comparisons of IPF vs. non-IPF subjects across these two datasets (p<0.001, false discovery rate (FDR) <0.001) (Figure 5), indicating that these genes were also up-regulated in IPF patients versus control subjects. The active fibrosis phase signatures were similarly up-regulated in IPF patients with acute exacerbations compared to more stable IPF subjects (p<0.001, FDR <0.01), suggesting that these signatures are capturing more active disease responses. Late fibrosis phase signatures (days 21–35) were also up-regulated in IPF patients vs. non-IPF control subjects from both clinical datasets (p<0.05, FDR <0.05). Inflammation phase response signatures, characteristic of initial immune response to bleomycin treatment, were generally not up-regulated in IPF subjects compared to controls.

**Figure 6:**
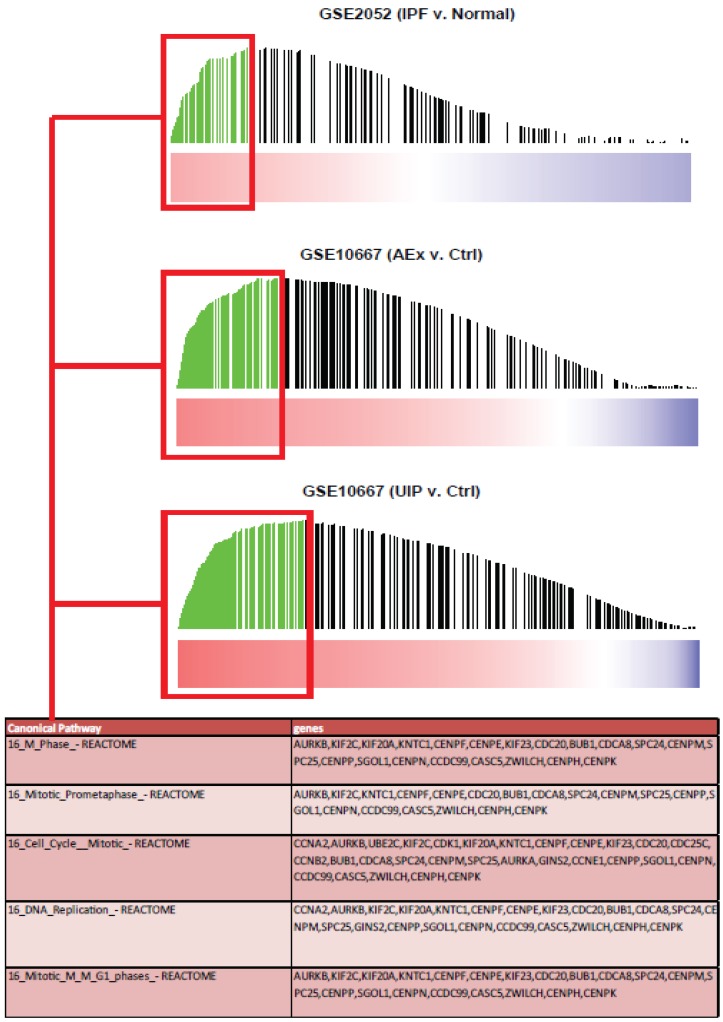
Analysis of leading edge gene subsets from GSEA of bleomycin-induced gene sets in IPF vs. non-IPF comparisons from two clinical cohorts (GSE2052, GSE10667). Red-blue color bars denote genes ranked based on fold-change and FDR in IPF vs. non-IPF comparisons within datasets. Each vertical bar corresponds to bleomycin-induced genes from the 14 day time point, with bar height corresponding to running enrichment score calculated in GSEA. Green bars correspond to genes in the leading edge subset, or those genes contributing most to enrichment of the gene set in human samples. A union of the leading edge gene subsets from the 3 enrichment analyses of IPF vs. non-IPF clinical samples was subsequently compared against canonical pathways to determine enriched pathways of bleomycin genes most altered in clinical IPF. The table illustrates the top 5 canonical pathways enriched among the leading edge gene subsets.

Closer examination of the leading edge genes (i.e. those genes that contribute most to the enrichment of the bleomycin signatures in the clinical fibrosis expression profiles) shows up-regulation of a common subset of genes across different clinical datasets (Figure 6). Genes in this leading edge subset form clusters characteristic of different processes associated with fibrosis (Data File S1, Data File S2). In particular, many cell cycle-related genes appear to dominate and include different cyclins, cell division cycle proteins (CDCs), and cyclin-dependent kinases (CDKs). Other fibrosis-related pathways such as platelet-derived growth factor (PDGF)-, integrin-, and syndecan-mediated signaling gene sets are also included within the leading edge. Additionally, we also found that (relative to their non-IPF controls) IPF patients’ lung sample gene expression profiles were enriched with genes associated with other fibrotic processes (i.e. MMPs, collagen, and the TGFβ signaling molecules) that we had also shown to be up-regulated in the bleomycin model (Figure S9). These results indicate that the genes associated with these pathways are commonly up-regulated in both bleomycin-induced and clinical fibrosis. As an attempt to “back-translate” signatures between clinical and *in vivo* datasets, GSEA also revealed that gene sets defined by changes in the clinical IPF datasets were enriched within the expression profiles of lung samples generated from the bleomycin model (data not shown). These results further highlight commonalities in genes altered in response to bleomycin treatment with those altered in clinical IPF samples.

### Mitotic Signatures Up-regulated in Fibroblasts from IPF Patients with Rapidly Progressing Disease

Next, we prospectively compared the gene expression profiles of fibroblasts obtained from patients with rapidly progressing and slowly progressing IPF to fibroblasts obtained from non-IPF control subjects, as these cells are centrally involved in the disease pathogenesis [1], [9]. As observed in the lung tissue profiles from our bleomycin model and the publicly available IPF datasets, mitotic processes were significantly increased in the fibroblasts from rapidly progressing IPF patients (Table 1), whereas the same cell cycle related processes were not altered in the fibroblasts from slowly progressing IPF patients relative to non-IPF control subjects. Among the top 25 significantly up-regulated canonical pathways from each comparison shown in Table 1 (i.e. rapidly progressing IPF versus non-IPF and day 14 post-bleomycin versus saline time-matched control), 16 were in commonly up-regulated in both systems. All 16 pathways involved cell cycle processes.

**Table 1:** Mitotic pathways are activated in both the bleomycin model and IPF patient fibroblasts.

Pathways commonly altered in both comparisons:	Fibroblasts - Rapid progressing IPF vs Non-IPF		Lung tissue - Bleomycin- vs Saline-treated	
*Canonical Pathways from Reactome*	*P.value-Fibroblast*	*FDR.value-Fibroblast*	*P.value- Bleo*	*FDR.value-Bleo*
ACTIVATION_OF_ATR_IN_RESPONSE_TOREPLICATION_STRESS	0	0	0	0
ACTIVATION_OF_THE_PRE_REPLICATIVECOMPLEX	0	0	0	0
CELL_CYCLE__MITOTIC	0	7.01E−05	0	0
CELL_CYCLE_CHECKPOINTS	0	0	0	0
CHROMOSOME_MAINTENANCE	0	0	0	6.78E−05
DNA_REPLICATION	0	0	0	0
DNA_REPLICATION_PRE_INITIATION	0	0	0	6.46E−05
DNA_STRAND_ELONGATION	0	0	0	0
EXTENSION_OF_TELOMERES	0	0.00107858	0	0.000833
G1_S_TRANSITION	0	0	0	0
G2_M_CHECKPOINTS	0	0.000983	0	0
M_G1_TRANSITION	0	0	0	0.000182
MITOTIC_G1_G1_S_PHASES	0	0	0	0
MITOTIC_M_M_G1_PHASES	0	0	0	0
S_PHASE	0	0	0	0
SYNTHESIS_OF_DNA	0	0	0	0

Overlapping enriched canonical pathways from gene set enrichment analysis of two studies: 1) cultured fibroblasts from rapid versus non-IPF subjects, and 2) lung tissue from bleomycin versus saline treated mice 14 days post-treatment. Nominal p-values and false discovery rates (FDR) indicate significance of enrichment of canonical pathways in each analysis.

### Effect of TGFβRI Inhibition in the Bleomycin Model

**Figure 7:**
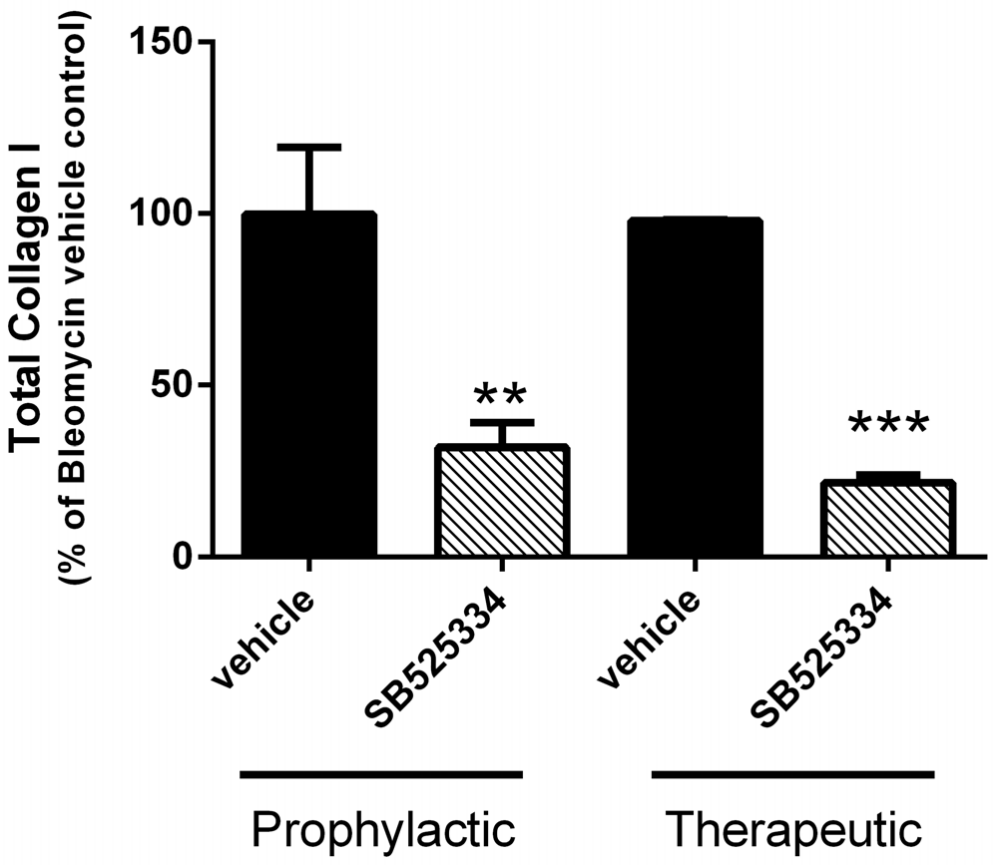
The ALK-5 inhibitor, SB525334, attenuates bleomycin-induced lung fibrosis. SB525334 was administered prophylactically (**A**) or therapeutically (**B**) at a dose of 60 mg/kg in chow beginning 1 day prior or 5 days after bleomycin instillation, respectively. SB525334 inhibited the lung fibrosis under both dosing conditions. Data are expressed as a percentage of the total collagen I stained area in the bleomycin vehicle control group ± SEM of n = 10–12 bleomycin treated mice in the prophylactic study and n = 6–9 in the therapeutic study. Saline treated control groups consisted of n = 5–7 mice. Significance (relative to the bleomycin vehicle control) was determined using a 2-tailed t-testand is denoted as **p<0.01 or ***p<0.001.

Given that several pathways associated with fibrotic processes were elevated in response to bleomycin-induced lung injury, we wanted to assess whether TGFβ was the dominant driver of fibrosis in this model. To this end, we assessed an ALK-5 inhibitor, SB525334, under both prophylactic and therapeutic conditions. SB525334 significantly inhibited bleomycin-induced fibrosis changes under both conditions (Figure 7A and 7B). The maximal effect we observed was approximately a 68±7% reduction in lung fibrosis when dosed prophylactically and a 78±2% reduction when dosed therapeutically. Of note, the dose used was twice as high as the dose that has been previously used to achieve maximal effects in other models of lung remodeling [4], [10]. Additionally, the plasma levels of SB525334 reached maximal levels 5 days after the introduction of the compound-chow diet (Figure S10). The peak plasma levels were clearly several fold above SB525334’s reported IC50 (∼30–100 nM) in a cellular assay [11].

## Discussion

The data presented in this manuscript provide clear evidence that the bleomycin model of lung fibrosis induces molecular changes directly relevant to IPF and in particular may be more relevant for modeling “active” rather than slowly progressing disease. Additionally, these data indicate that there are multiple pathways involved in eliciting the pathophysiological changes in the model that are consistent with those observed in the lungs of patients with IPF. As such, it would appear that therapies aimed at affecting several of these key signaling networks either by targeting “nodes”, using combination therapy approaches, or broad spectrum inhibitors of key enzymes may be the only means of making a significant impact on disease progression in all patients with IPF.

The response to bleomycin occurred in three distinct phases, which we have termed the (1) inflammation phase, (2) active fibrosis phase, and (3) late fibrosis phase. The inflammation phase was largely characterized by the presence of neutrophils in the airways and the increased expression of immune processes in the lung tissue. While some have proposed that neutrophils may be playing a role in IPF [12], it is clear from our data that the molecular changes occurring during this phase of the response did not correlate to molecular changes observed in the lung tissues of IPF patients. These observations would support the hypothesis posed by others [3] that targeting mechanisms occurring during this phase of the response are not likely to translate into a therapeutic benefit in the clinic. Thus, the testing of candidate therapies prophylactically in this model may have provided misleading data that resulted in several failed clinical trials – the major criticism of the bleomycin model.

Another commonly stated limitation of the bleomycin model is that the fibrosis is not progressive and in fact, resolves with time. Interestingly, we observed that there was still measureable fibrosis and changes in pulmonary mechanics in the late fibrosis phase (out to 5 weeks post-bleomycin). Furthermore, we took samples 56 days after bleomycin instillation in a separate study (data not shown) and measureable fibrosis was still present contrary to previous reports and dogma [13]. That said, one limitation of this model is the fact that the fibrosis is not progressive and as such, the impact the fibrosis has on pulmonary mechanics is limited. While we can consistently observe changes in lung tissue elastance (*H*) and our new measure of WoI [14] during the fibrotic phases of the response to bleomycin, the changes are not as substantial as in the human disease and do not appear to get worse with time [15]. The lack of substantial changes in lung mechanics is likely due to the significant reserve functional capacity of rodents’ lungs. As a way of pushing the system, we chose to measure exercise capacity (running on a treadmill), which showed some promise for longitudinally tracking progressive changes in the physiological functioning of the animals. This method allowed us to assess the maximum capacity of the cardio-respiratory system, which obviously takes more than pulmonary function into account; however, it may provide great utility because it is clinically relevant, non-invasive, and allows investigators to select and subsequently randomize animals into treatment groups. The latter may improve the design of efficacy studies given the fibrosis (and hence functional change) induced by bleomycin appears to progress at slightly different rates amongst animals; an observation that was also evident from the hierarchical clustering analysis of the microarray data.

As an attempt to improve the single challenge system we performed repetitive bleomycin instillations, but found that repetitive exposures provided no additional value in terms of producing a more substantial or progressive fibrotic response in the lung. The one difference to the single challenge model was that epithelial remodeling was more evident at most of the time points assessed in the repetitive challenge system. Epithelial remodeling in IPF is associated with the deterioration of the lung parenchyma and the honeycombing pattern of the lung characteristic of IPF [16]. That said, these changes were still observable in the single challenge model especially at later time points (i.e. day 35). At present, we cannot explain the differences between the data we report to those reported by Degryse and colleagues who first described this chronic bleomycin model protocol [6]. It is possible that a slightly different method of delivery (i.e. our use of a microspray device) or source of bleomycin may be a reason for some of the differences observed. That said, given the laborious nature of the repetitive model, our data would indicate that there is no significant advantage to using this system over the single challenge model.

The most interesting observations from these studies occurred during the active fibrosis phase of the response to bleomycin. This phase was characterized by increased levels of (protein and lipid) pro-fibrotic mediators associated with increased matrix deposition in the lung that correlated with alterations in pulmonary mechanics. These changes were also temporally correlated with the increased expression of a variety of fibrosis-related gene sets including TGFβ signaling, matrix proteases, and structural proteins – observations that confirm previous findings reported by others [17]–[19].

Here, we have shown for the first time a direct correlation between the molecular changes observed in the lungs of bleomycin-treated mice and patients with IPF using novel translational and quantitative bioinformatics approaches. GSEA allows one to define gene signatures from one experimental system and subsequently quantify whether that signature is enriched in another biological system. The gene sets defined by changes during the active fibrosis phase (days 7–14) of the mouse bleomycin model generated the highest enrichment scores amongst the samples from IPF patients obtained from two separate publicly available data-sets. In fact, the changes occurring in the late fibrosis phase were also enriched in the clinical samples, an observation that is consistent with the lack of fibrosis resolution at these later times. To understand the biology behind the correlative gene changes, we overlapped a union of the leading edge gene lists with canonical signaling pathways. Interestingly, the leading edge genes were largely involved in mitotic processes likely due to the proliferation of mesenchymal cells. Additionally, there were changes in signaling pathways (PDGF, syndecans, and integrins) associated with fibroblast proliferation and signaling in response to changes to the extracellular matrix. These data highlight the multifactorial molecular mechanisms involved in the pathogenesis of IPF [1], [9], [20].

Although these are interesting observations, additional clinical information such as lung function data, high resolution computed tomography scores, and rate of disease progression associated with publicly available clinical datasets would improve the interpretation of these datasets. Such information would provide greater insights about whether these molecular changes that correlate with the model are generic amongst all IPF patients or whether the correlation is strongest within certain IPF patient sub-populations. Interestingly, many of the overlapping changes between the model and clinical samples were likely to be due to changes in fibroblast activation and proliferation. These same processes were enriched in fibroblasts isolated from the lungs of IPF patients with rapidly progressing disease. Surprisingly, these processes did not appear to be altered when comparing fibroblasts from patients with slowly progressing IPF to non-IPF controls. Together, these observations would suggest that the bleomycin model may be more effective at mimicking active disease processes that may be more associated with patients with a rapidly progressing disease course. That said, there are caveats to these data that need to be taken into account. First, the patients’ cells are not freshly isolated or laser capture microdissected, but rather they have been in culture for several passages, which may confound interpretation. Second, the controls are not healthy individuals, but cancer patients undergoing lung resection. Finally, these data were generated from 4–6 patients within each group and additional biological replicates would improve confidence in these observations. That said, it is still very interesting to note that the cells from the rapidly progressing IPF patients do have enhanced responses to PDGF (proliferation, migration) and TGFβ (contraction, cytokine production), whereas these responses are not as profoundly altered in fibroblasts from slowly progressing IPF patients relative to the non-IPF controls (*Budd et al, under review*). Additionally, the cells from rapidly progressing patients are able to induce substantial lung fibrosis when injected into immunocompromised mice (versus a mild response when using slowly progressing IPF patient fibroblasts) [21], indicating that the cells from these patients retain a phenotype with greater pathogenic potential.

Another interesting observation worth noting was from the GSEA of the TGFβ, protease, and matrix protein gene sets in the clinical data-sets. Unlike the protease and matrix protein gene sets that were consistently up-regulated in all of the IPF patient data-sets, the TGFβ up-regulated gene set (comprised mainly of positive regulators of the TGFβ pathway) was only enriched in the IPF patients who had experienced an acute exacerbation compared to non-IPF control subjects, possibly indicating its importance in active disease. Similarly, the down-regulated TGFβ gene set (comprised mainly of negative regulators of the TGFβ pathway) was only down-regulated in one clinical data-set. Although no one would discount TGFβ’s contribution to the lung remodeling associated with IPF, it does highlight the fact that it is unlikely to be the only pathway driving disease pathogenesis. These observations are also consistent with the data demonstrating that the ALK-5 inhibitor, SB525334, was not capable of fully blocking the fibrotic response elicited by bleomycin. This molecule consistently attenuates the response in our laboratory as well as in others [4], and the inability to completely inhibit the fibrotic response is consistent with other genetic approaches that have targeted key elements of the TGFβ signaling pathway [22], [23]. Collectively, these data suggest that additional pathways [24] are also likely to contribute to the IPF-like pathologies that develop in this model, which is consistent with our observations that other pro-fibrotic mediators (e.g. LPA) and pathways (e.g. Wnt, PDGF) are up-regulated during the fibrotic phases of the response to bleomycin. Therefore, one question posed by these data: How much of an impact on lung fibrosis does a molecule need to achieve in this model in order for the effect to be observable clinically?

The data described in this paper contradict current dogma and suggest that bleomycin is able to induce clinically meaningful molecular responses in the lungs of mice. Using innovative bioinformatic approaches we directly linked the molecular changes occurring in the bleomycin model to those occurring in the lungs of IPF patients in a quantitative manner. Genes involved in mitotic processes were expressed at higher levels in lung tissues from bleomycin-treated mice and IPF patients as well as fibroblasts isolated from IPF patients with rapidly progressing disease. These data along with pharmacological data indicate that bleomycin triggers complex, multifactorial (TGFβ dependent and independent) responses that result in pathophysiological changes in mice that are consistent with IPF. Furthermore, our analysis suggests that the changes in this model best represent changes occurring in patients with active (i.e. rapidly progressing) disease.

## Materials and Methods

### Animals

Male C57BL6/J mice (7–9 weeks old) were purchased from the Jackson Laboratory (Bar Harbor, ME), housed under pathogen-free conditions with food and water *ad libitum*. All animal care and experimental procedures were approved by the Roche Animal Care and Use Committee, which is a facility accredited by the American Association for the Accreditation of Laboratory Animal Care (AAALAC).

### Administration of Bleomycin

Bleomycin sulphate (MP Biomedicals, Solon, OH, USA) was dissolved in 0.9% saline and loaded into a IA-1C liquid MicroSprayer (PennCentury, Wyndmoor, PA, USA) as previously described [25]. For dose response studies bleomycin (1–5 U/kg) in 50 μl was intratracheally sprayed into mice lightly anaesthetized with isoflurane (5% in 100% O2). Control animals received 50 μl of 0.9% saline. For the single challenge model time course study, animals were administered bleomycin (2 U/kg) once. For the repetitive challenge time course study, animals were administered bleomycin (2 U/kg) every other week for a total of 8 doses. For efficacy studies, the normal chow diet was replaced with Purina Rodent Chow #5001 mixed with SB525334 starting on 2 days (prophylactic) or 5 days after (therapeutic) bleomycin administration (2 U/kg). The formulation contained 999.200 g of Purina Rodent Chow #5001, 0.5 g of SB525334, and 0.3 g FD&C Red Dye #40 to achieve a daily dose of 60 mg/kg. Control groups received Purina Rodent Chow #5001. Animals remained on this diet every day until the end of the study (day 21 post-bleomycin administration).

### Lung Function

Invasive lung function measurements were taken at specified time points following saline or bleomycin administration as previously described [25]. Briefly, mice were anesthetized with 2.5 mg sodium pentobarbital (Abbott Labs, IL), and the trachea cannulated. Animals were mechanically ventilated using a computer-controlled piston ventilator (flexiVent, SCIREQ Inc., Montreal, Canada; tidal volume = 10 ml/kg, respiratory rate of 150 breaths/min, 3 cmH_2_O positive end-expiratory pressure).

### Measuring Exercise Capacity

Exercise capacity was measured based on a method previously published by Koch and colleagues [26] with minor modifications. Briefly, animals were trained on the treadmill a week prior to the first exercise test. To test exercise capacity animals were placed on a treadmill at a 5% incline at a speed to 10 m/minute and the speed was increased 1 m/minute every 4 minutes of the test until exhausted, which was defined as the third consecutive time the animal fell onto the stimulus grid for more than 2 seconds. Baseline exercise capacity for each mouse was assessed 2 days prior to either saline or bleomycin administration and then once weekly after administration at days 6, 12, and 19 post-instillation.

### Bronchoalveolar Lavage Cell and Mediator Assessment

Immediately following lung function measurements, mice were exsanguinated, bronchoalveolar lavage fluid (BALF) was collected, and total and differential cell counts were performed, as previously described [25]. Samples were centrifuged (300×g) and the remaining BALF supernatants were stored at −80°C until analyzed for cytokine and chemokine levels using MSD multiplex kits (Gaithersburg, MD) and Millipore immunoassay kits (Billerica, MA) following the manufacturer’s protocol. An LC-MS/MS method was developed for the analysis of Lysophosphatidic Acid (LPA) forms (i.e. 16:0, 18:0, 18:1, and 20:4) in BALF. The standard addition spike-in range is 10–500 ng/mL into blank BALF. The extraction method consists of a liquid-liquid extraction of ionization-interfering matrix components into MtBE and away from the BALF sample which is then extracted by protein-precipitation with methanol. Samples are directly injected onto a reverse phase HPLC column for separation of the analytes, which are then introduced to the mass spectrometer by electrospray ionization, and analyzed by MS/MS. Standard addition determinations of the individual endogenous LPA concentrations are made, followed by correction of the standard curve, and finally quantitation of the individual sample concentrations using linear, 1/×2 regression.

### Assessment of BALF Hydroxyproline Levels

For the analysis of 4-hydroxyproline (HP), BALF samples were extracted with acetonitrile at volume of 1:6, respectively. After centrifugation, acetic acid (0.1% in water) was then added to the supernatants at volume of 1.5:1, respectively. Samples were mixed and centrifuged before injection for LC/MS/MS analysis. LC/MS/MS analysis was conducted by gradient HPLC with selective reaction monitoring (SRM). The calibration range was 40- 2,000 ng/ml. Since the control BALF matrix contained a basal level of HP (approximately 200 ng/mL), the calibration curve is corrected with the basal concentration determined by the standard addition. The LC/MS/MS system used for the analysis consisted of an AB-Sciex API4000 with an electrospray source connected to an Agilent 1200 pump, an Waters 2777 autosampler, and an Agilent 1100 series column oven. Chromatographic analysis was conducted by HILIC HPLC with an Ascentis Express HILIC (30×2.1 mm, 2.7 um) column. The SRM transition is m/z 132 to m/z 41.

### Histology

After performing lung function measurements, whole lungs (that were not subject to bronchoalveolar lavage) were inflated under 25 cm H_2_O pressure with 10% neutral buffered formalin through the tracheal cannula and immersed in formalin for at least 24 h. After being processed into paraffin blocks, the lungs were sectioned (5 µm) and stained with either hematoxylin and eosin (H&E) or immunolabeled with an anti-collagen I antibody (rabbit polyclonal, Genetex Inc., Irvine, CA), to assess fibrotic changes in the lungs as previously described [25], [27], with minor differences. To determine the fibrosis histopathology score for the lung of each mouse, the entire left and right longitudinal lung sections (at the level of tracheal bifurcation) were scored separately at 100X magnification and the scores were combined (total score range 0–8). Grading criteria were as follows: Grade 0 = no apparent fibrosis; Grade 1 = minimal fibrosis with rare foci of mostly interstitial alveolar septal fibrosis affecting less than 5% of the entire lung section; Grade 2 = mild fibrosis characterized by multiple foci with thickening of alveolar septa by fibrosis and progressing to regions with fibrous deposition within the alveolar spaces with some damage to the alveoli, affecting 5–25% of the entire lung section; Grade 3 = moderate fibrosis with multiple or single coalescing large areas of fibrosis effacing the alveoli with definitive damage to pulmonary architecture, affecting 25–50% of the entire lung section; Grade 4 = marked fibrosis with severe distortion of pulmonary parenchyma by large contiguous fibrous areas, affecting 50–75% of the entire lung section; Grade 5- massive pulmonary fibrosis similar to Grade 4 affecting 75–100% of the entire lung section. In these studies, there were no samples with fibrosis that warranted a score of Grade 5; therefore, the scores are reported as being from 0–8 for each study. For collagen I IHC quantification, glass slides were scanned at 20X using a Zeiss Mirax scanner (Carl Zeiss Microimaging, Thornwood, NY), and digital slides generated were analyzed using Definiens Tissue Studio software (Definiens, Munich, Germany). For the analysis we developed a computer-based algorithm to automatically identify only parenchymal lung areas for analysis and quantification of collagen I immunolabeling in areas with fibrosis. Numerical results were expressed as percentage of positively-labeled area (area of collagen I labeling/parenchymal tissue reference area).

Additionally, staining for alpha-smooth muscle actin (Thermo Scientific, Freemont, CA), and heat-shock protein-47 (HSP-47) (clone EPR4217, Epitomics, Burlingame, CA) was done using a 3-step biotin-streptavidin-HRP detection method, and DAB (3,3-diaminobenzidine) as the detection chromogen. Tissue sections were counterstained with hematoxylin, dehydrated, and mounted with Permount mounting media.

### Genomic Profiling of Bleomycin and Saline Treated Mouse Lung Tissue

Total RNA was isolated from the lung tissue of bleomycin and saline treated mice across 7 time points (1, 2, 7, 14, 21, 28, and 35 days, n = 8 per group). The mice used for genomic profiling were not used for measuring lung function, performing bronchoalveolar lavage, and they were not used for histopathology analysis. The entire left and right lung tissues were homogenized in QIAzol reagent. Purified total RNA was amplified and labeled using NuGen Ovation kits (NuGEN Technologies, Inc., San Carlos, CA) and samples were hybridized to Affymetrix Mouse 430 2.0 arrays. Array washing, staining and scanning was performed according to standard Affymetrix protocols (Affymetrix Inc., Santa Clara, CA). Probe level data was curated by first mapping individual probe sequences to their most current mouse genome sequences. Probes which were non-uniquely mapped to specific genes or contained outdated mappings were discarded, and the remaining probes were summarized into probesets and normalized using RMA. Potential outlier samples were assessed using principal component analysis (PCA) on all normalized probesets across all samples, resulting in 1 sample (saline, day 14) being removed from subsequent analysis. Probes level data was subsequently summarized to unique genes based on a variance filter, yielding one expression value per unique gene across 111 samples. Mouse genes were then mapped to their human orthologs for subsequent pathway analysis. This resulted in 14643 unique mouse genes which mapped to human orthologs and were subsequently used for analysis. All data is MIAME compliant and all raw.CEL files from the arrays are deposited in NCBI’s GEO database (GSE40151).

Differentially expressed genes were determined using an ANOVA, with pairwise comparisons between bleomycin and saline treatment at each time point. P-values for differentially expressed genes in pairwise comparisons were adjusted using a Benjamini-Hochberg correction to account for multiple hypothesis testing [28]. Genes that were significantly altered at least 2-fold between bleomycin treatment and saline controls (False discovery rate, FDR <0.05) were considered to be differentially expressed. Unsupervised hierarchical clustering was performed on the union of differentially expressed genes (DEGs) between bleomycin and saline-treated samples across all time points to determine phases of response to bleomycin treatment. Common and unique genes between these phases of response were determined for each phase by taking the union and intersection of differentially expressed genes. Unsupervised hierarchical clustering was performed on a union of DEGs between bleomycin and saline treatments across all time points. A total of 730 genes were clustered across all mouse samples to determine phases of genomic response to bleomycin treatment and links between responses observed at the gene expression and protein level.

### Gene Ontology and Pathway Analysis

Gene ontology (GO) functional analysis and pathway enrichment analysis were performed on DEGs between saline and bleomycin treated mice at each time point using internally curated GO biological process annotations. Similarly, pathway enrichment for all DEGs was determined using internally curated data from the NCI Pathway Interaction Database (http://pid.nci.nih.gov/index.shtml). This repository includes pathway data imported from BioCarta and Reactome. Enrichment of functional ontologies and pathways for all DEGs was determined by a hypergeometric test, with p-values and FDR calculated to determine significance of enrichment.

Additional sets of genes from representative gene families or pathways of interest were examined in expression data from bleomycin and saline treated mice using supervised hierarchical clustering to determine sets of genes within pathways or gene families which were commonly expressed across samples. Pathways and gene families considered included metalloproteases (MMPs, ADAMs), collagens (COLs), TGFβ signaling (a gene set manually curated from the literature), alternative macrophage activation, Wnt signaling, and an *in vitro* PI3-kinase gene set from a previous study [29].

### Gene Set Enrichment Analysis of Custom Inflammatory Gene Signatures

Gene set enrichment analysis (GSEA) [29] was used to determine enrichment of in vivo bleomycin signatures in two clinical IPF gene expression datasets available in the Gene Expression Omnibus (GEO): GSE2052 [7] and GSE10667 [8]. Comparisons involving IPF subjects within each clinical dataset were used to determine whether genes altered in response to bleomycin treatment in mice were also similarly changed in human subjects with stable IPF and acute exacerbations of IPF compared to non-diseased controls. Briefly, enrichment of bleomycin gene sets was calculated against all genes within each clinical IPF dataset, ranked based on a composite score of fold-change and FDR differences between disease subgroups within each dataset. FDR values were determined for gene set enrichment by permuting genes within gene sets.

Clustering analysis was subsequently performed on leading edge genes from GSEA, in order to more closely examine genes within bleomycin signatures most contributing to enrichment of the signatures in clinical datasets. A union of leading edge genes from GSEA of IPF versus control comparisons from two datasets (GSE2052 and GSE10667, in which IPF subjects had no other known co-morbidities) was compiled and expression of these genes was assessed in the two clinical datasets with IPF-only subjects versus controls.

In addition, GSEA was used to determine enrichment of genes differentially expressed in clinical IPF datasets in bleomycin versus saline treated mice. DEGs were determined in each IPF dataset between IPF and non-IPF groups (IPF vs. normal from GSE 2052, UIP vs. Control and Acute exacerbators vs. Control from GSE10667). DEGs were determined based on being 1.5-fold altered between groups with an FDR <0.05. A union of DEGs from comparisons in each dataset was compiled resulting in two gene sets consisting of 30 genes (21 up-regulated in IPF, 9 down-regulated in IPF). Enrichment of these gene sets was determined against genes from each time point in the mouse bleomycin data, ranked based on a composite of fold-change and FDR differences between bleomycin and saline treated samples per time point, with FDRs of gene set enrichment determined as described above.

GSEA was also used to determine enrichment of internally curated canonical pathways from the NCI Pathway Interaction Database in both the mouse bleomycin and cultured human fibroblast datasets. Canonical pathways enriched in the bleomycin dataset were defined by ranking genes based on a composite score of fold-difference and FDR between bleomycin and saline-treatments for the 14 day time point (active fibrosis phase). Canonical pathways were then used as gene sets for GSEA, with FDR values being determined for gene set enrichment by permuting genes. Canonical pathway enrichment was similarly determined in the fibroblast dataset by generating ranked gene as described above in rapid progressing IPF vs. non-IPF samples. Significantly enriched canonical pathways (p<0.05, FDR <0.05) were then compared between the bleomycin-treated 14 day time point and fibroblasts from rapid progressing IPF patients.

### Genomic Profiling of Cultured Fibroblast Cell Lines

Primary cultures of LFs were isolated from the distal parenchyma of patients from unaffected donor controls (lung cancer patients) and patients with IPF as previously described [30], [31]. All fibroblast cell lines were completely de-identified from the patients they were grown from prior to characterization in this study. The IPF lung tissue was obtained and the diagnosis of Usual Interstitial Pneumonia (UIP)/IPF achieved as previously outlined [31]. Normal human lung tissue was obtained from macroscopically tumor-free lung resections of patients with lung cancer. The human lung tissue collection was approved by the ethics committees of all institutions involved. In this study we characterized fibroblasts cell lines originated from 14 donors across 3 phenotypes: non-IPF control (n = 4), stable IPF (n = 6), and rapidly progressing IPF (n = 4) subjects. Cells from these lines were plated and passaged (up to the 11th passage). Messenger RNA from harvested cells from each cell line across these phenotypes was isolated and homogenized in QIAzol reagent. Purified RNA was then amplified and labeled using NuGen Ovation kits (NuGEN Technologies, Inc., San Carlos, CA), and subsequently hybridized to Affymetrix HG-U133 plus 2.0 microarrays. Array washing, staining and scanning was performed according to standard Affymetrix protocols (Affymetrix Inc., Santa Clara, CA). Probe level data was curated by first mapping individual probe sequences to their most current genome sequences and removing probes expressed below a defined background expression level. Probes which were non-uniquely mapped to specific genes or contained outdated mappings were discarded, and the remaining probes were summarized into probesets and normalized using RMA. Probes level data was subsequently summarized to unique genes based on a variance filter, yielding one expression value per unique gene across all 14 samples. This resulted in 12018 unique genes used for subsequent analysis. All data is MIAME compliant and all raw.CEL files from the arrays are deposited in NCBI’s GEO database (GSE44723).

### Additional Statistical Analysis

Data are expressed as mean ± SEM unless specified otherwise. Unless stated otherwise elsewhere in the methods, statistical analyses of time course studies were determined using a Student’s t-test using GraphPad Prism (version 5.03).

## Supporting Information

Figure S1 **Bleomycin induces dose-dependent increases in lung fibrosis.** Fibrosis scores evaluated in H&E stained lung sections 21 days after a single instillation of bleomycin. Data expressed as mean ± SEM of n = 8 except for the 5 U/kg group where n = 5. Significance relative to the saline treated was determined using a one-way ANOVA and Dunnett’s post-hoc test and is denoted as, *p<0.05; or ***, p<0.001.
(TIF)

Figure S2 **Bleomycin induces inflammatory cell infiltrate in the airways of mice.** The numbers inflammatory cells increase in the BALF fluid after a single (**A** – **C**) or repetitive (**D** – **F**) bleomycin (black bars) or saline (white bars) administration. Differential cell counts revealed that neutrophils (**A** and **D**), lymphocytes (**B** and **E**), and macrophages (**C** and **F**) were all elevated in the BALF. Data are expressed as mean ± SEM of n = 7–8 mice. Significance (relative to the time-matched control at each time point) was determined using a Student’s t-test and is denoted as follows: *p<0.05; **p<0.01; and ***p<0.001.
(TIF)

Figure S3 **Bleomycin induces collagen deposition in the lung.** Measureable changes in matrix remodeling were observable after a single (**A** and **B**) or repetitive (**C** and **D**) bleomycin (black bars) or saline (white bars) administration. Lung collagen (**A** and **C**), and BALF hydroxyproline levels (**B** and **D**) were all elevated in response to bleomycin. Data are expressed as mean ± SEM of n = 7–8 mice. Significance (relative to the time-matched control at each time point) was determined using a Student’s t-test and is denoted as follows: *p<0.05; **p<0.01; and ***p<0.001.
(TIF)

Figure S4 **Changes in lung mechanics correlate to changes in lung fibrosis.** Changes in *H* (**A**) and volume normalized work of inflation (WoI) (**B**) after a single bleomycin instillation significantly correlated to the amount of lung tissue stained positive for collagen I as determined by Pearson correlation coefficients (R). The correlations for *H* (**C**) and WoI (**D**) with collagen I were similar after repetitive bleomycin instillations.
(TIF)

Figure S5 **Bleomycin increased collagen deposition and myofibroblast activation.** Serial lung tissue sections from one representative animal illustrate that areas positive for collagen I (**D**) were also positive for α-smooth muscle actin (**E**), as well as HSP47 (**F**). As a reference, sections from saline treated animals stained for collagen I (**A**), α-smooth muscle actin (**B**), and HSP47 (**C**) are also shown. Images were captured at 200X magnification.
(TIF)

Figure S6 **Exercise capacity is reduced in bleomycin treated mice.** The exercise capacity of mice treated with saline (filled circles) or 2 U/kg bleomycin (filled squares) was measured on a motorized treadmill. Data expressed as mean ± SEM of n = 19–20. Significance relative to the saline treated was determined using a one-way ANOVA and Dunnett’s post-hoc test and is denoted as, *p<0.05; or ***, p<0.001.
(TIF)

Figure S7 **Bleomycin induces cytokine, chemokine, and pro-fibrotic mediator secretion in the BALF.** BALF levels of KC (A), IL-16 (B), CCL17 (TARC) (C), total IL-12 (D), TGFβ (E), and LPA (18:1 isoform) (F)were measured. Saline treated controls represented by white bars and bleomycin treated animals by black bars. The following cytokines had no measurable change: IFN-g, TNF-α, IL-1β, IL-2, IL-4, IL-5, IL-10, GM-CSF, VEGF, IL-13, IL-17 (data not shown). Data are expressed as mean ± SEM of n = 8 mice. Significance (relative to the time-matched control at each time point) was determined using a Student’s t-test and is denoted as, *p<0.05.
(TIF)

Figure S8 **Supervised hierarchical clustering of custom panels of genes across all mouse samples.** Clustering of genes was performed for a panel of (**A**) genes involved in Wnt signaling, (**B**) genes altered downstream of PI3 kinase, and (**C**) genes involved in alternative macrophage activation**. S**amples are ordered based on treatment and time point.
(TIF)

Figure S9 **Heatmap of gene set enrichment of custom signatures in clinical IPF datasets.** GSEA was performed for gene sets of custom gene panels from fibrosis-related mechanisms including MMP, LOXL, collagen, and TGFβ signaling. Enrichment of each gene set (denoted in rows) was determined against ranked lists of genes from clinical datasets comparing IPF vs. non-IPF conditions from two datasets (GSE2052, GSE10667, denoted in columns). Enrichment scores were plotted in a heatmap where gene sets enriched in IPF samples (nominal p<0.05, FDR <0.25) were denoted in red while gene sets enriched in non-IPF samples were denoted in blue. Intensity of each cell was based on enrichment score (calculated in GSEA).
(TIF)

Figure S10 Plasma exposure levels for SB525334 in bleomycin treated mice after initiating a diet using Purina Rodent Chow #5001 mixed with SB525334.
(TIF)

Data File S1 **A union of leading edge genes from GSEA of bleomycin-induced gene sets in IPF vs. non-IPF subject comparisons from two clinical cohorts (GSE2052, GSE10667).** This alphabetized list of genes corresponds to a union of the genes highlighted in Figure 6.
(PDF)

Data File S2 **Canonical pathways enriched among the leading edge genes from GSEA of bleomycin-induced gene sets in IPF vs. non-IPF subject comparisons from two clinical cohorts (GSE2052, GSE10667).** The canonical pathways, p value of the enrichment of leading edge genes in these pathways, and the leading edge genes contained within the pathways are highlighted in this table.
(PDF)
