# Supplementary figures and images for: Bleomycin Induces Molecular Changes Directly Relevant to Idiopathic Pulmonary Fibrosis: A Model for “Active” Disease
Source: PLoS One. 2013 Apr 2;8(4):e59348. doi: 10.1371/journal.pone.0059348 (PMC3614979; doi:10.1371/journal.pone.0059348)

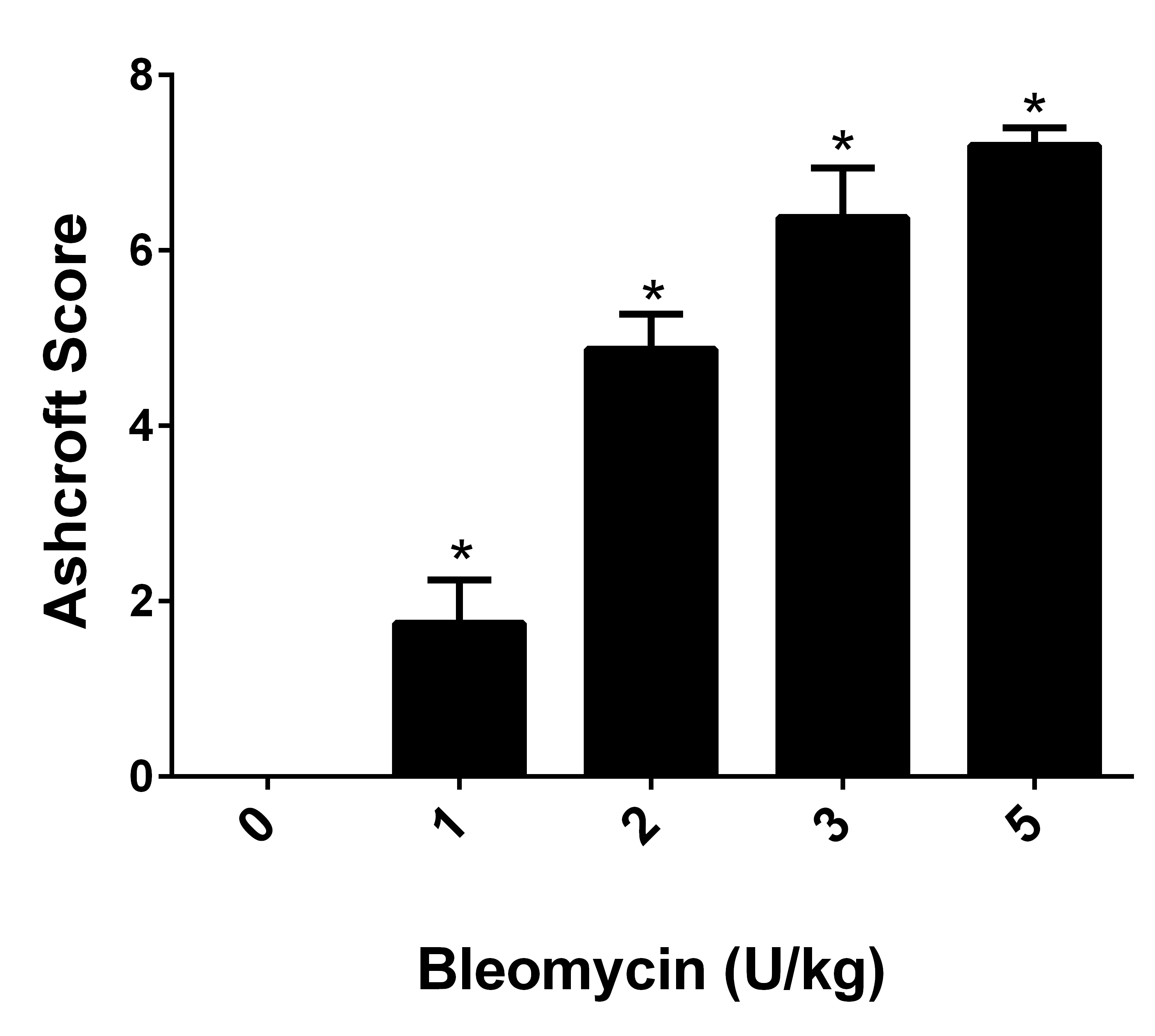

Supplement: Figure S1 — Bleomycin induces dose-dependent increases in lung fibrosis. Fibrosis scores evaluated in H&E stained lung sections 21 days after a single instillation of bleomycin. Data expressed as mean ± SEM of n = 8 except for the 5 U/kg group where n = 5. Significance relative to the saline treated was determined using a one-way ANOVA and Dunnett’s post-hoc test and is denoted as, *p<0.05; or ***, p<0.001. (TIF) [file pone.0059348.s001.tif]

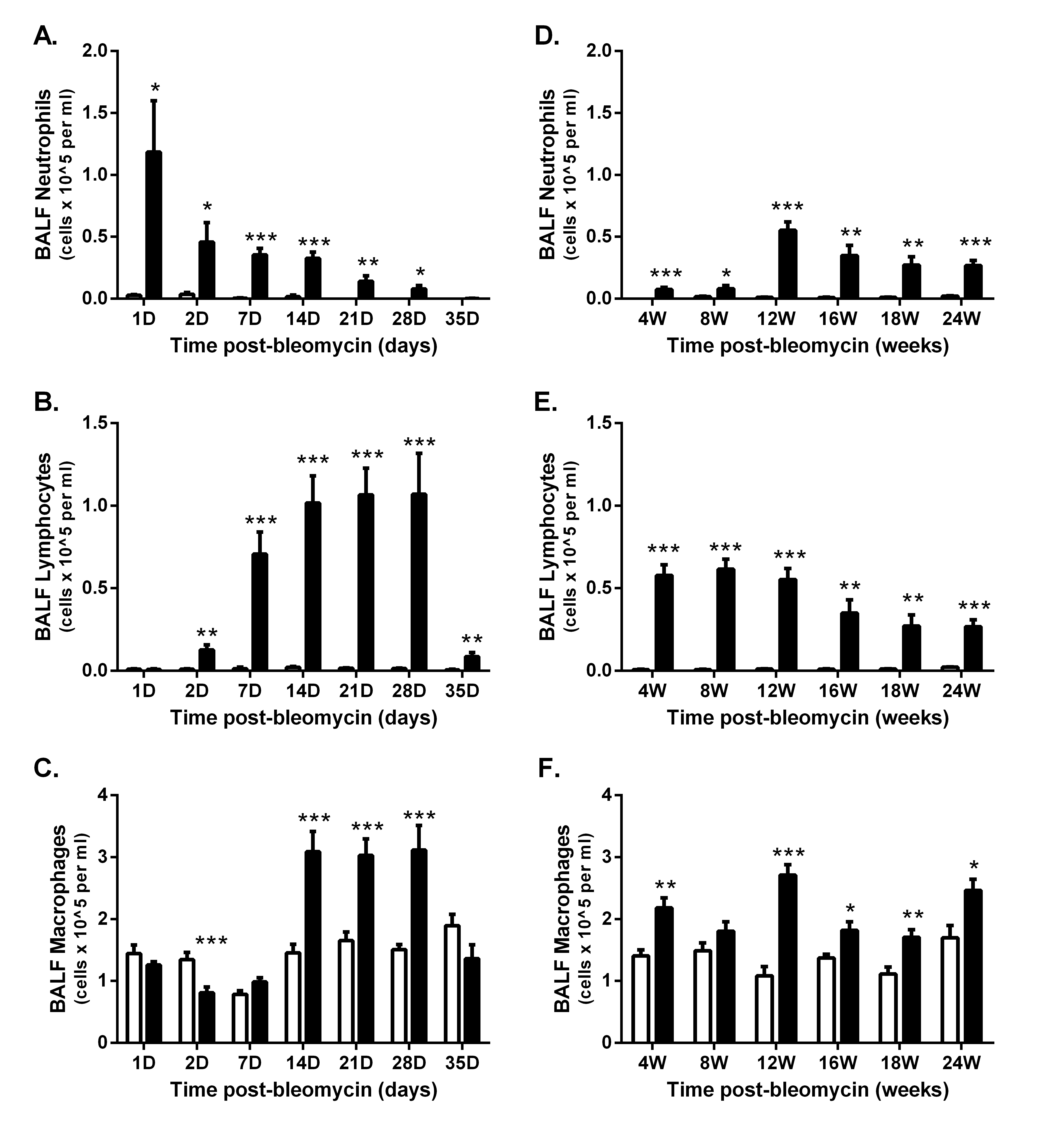

Supplement: Figure S2 — Bleomycin induces inflammatory cell infiltrate in the airways of mice. The numbers inflammatory cells increase in the BALF fluid after a single (A – C) or repetitive (D – F) bleomycin (black bars) or saline (white bars) administration. Differential cell counts revealed that neutrophils (A and D), lymphocytes (B and E), and macrophages (C and F) were all elevated in the BALF. Data are expressed as mean ± SEM of n = 7–8 mice. Significance (relative to the time-matched control at each time point) was determined using a Student’s t-test and is denoted as follows: *p<0.05; **p<0.01; and ***p<0.001. (TIF) [file pone.0059348.s002.tif]

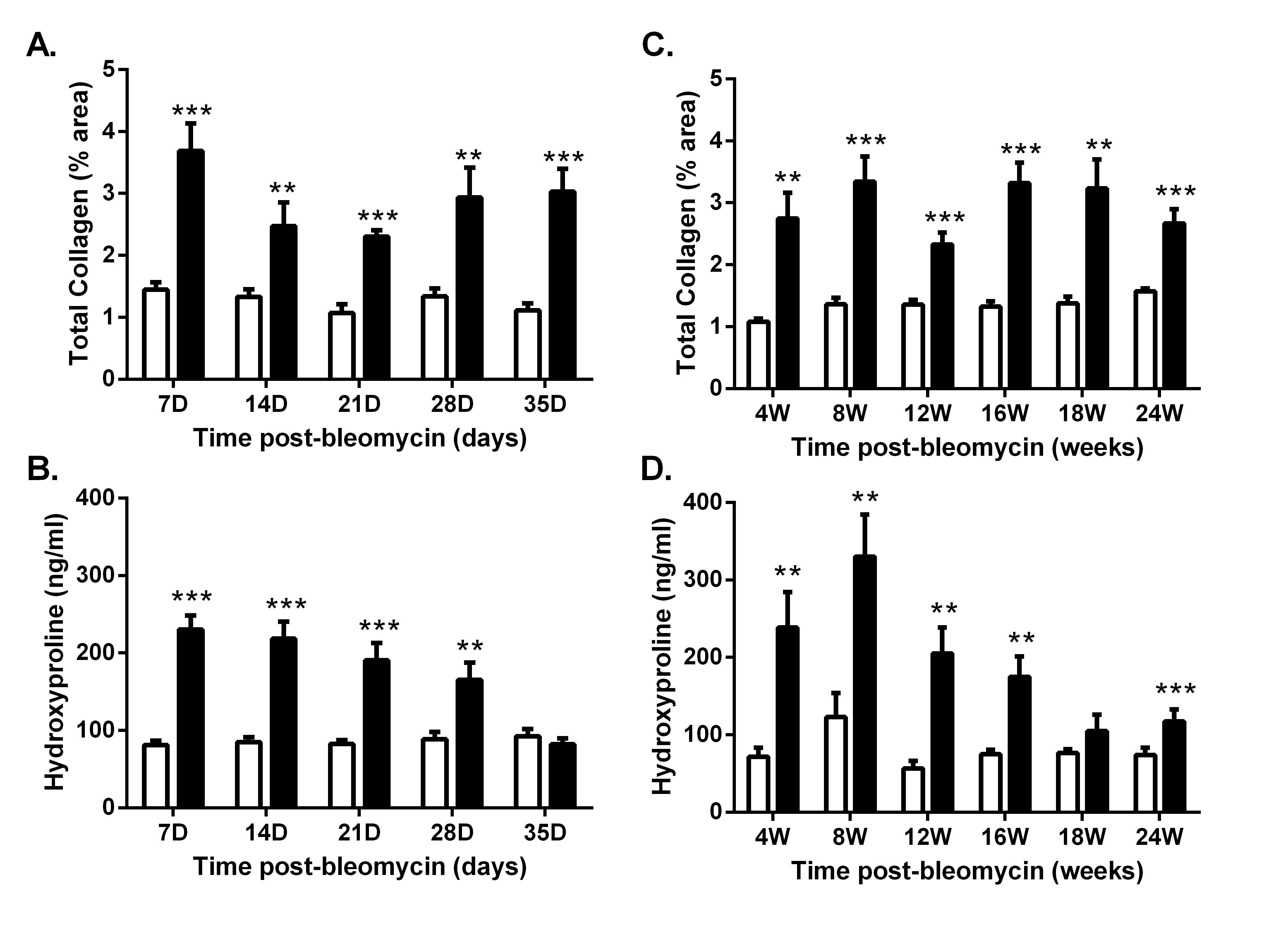

Supplement: Figure S3 — Bleomycin induces collagen deposition in the lung. Measureable changes in matrix remodeling were observable after a single (A and B) or repetitive (C and D) bleomycin (black bars) or saline (white bars) administration. Lung collagen (A and C), and BALF hydroxyproline levels (B and D) were all elevated in response to bleomycin. Data are expressed as mean ± SEM of n = 7–8 mice. Significance (relative to the time-matched control at each time point) was determined using a Student’s t-test and is denoted as follows: *p<0.05; **p<0.01; and ***p<0.001. (TIF) [file pone.0059348.s003.tif]

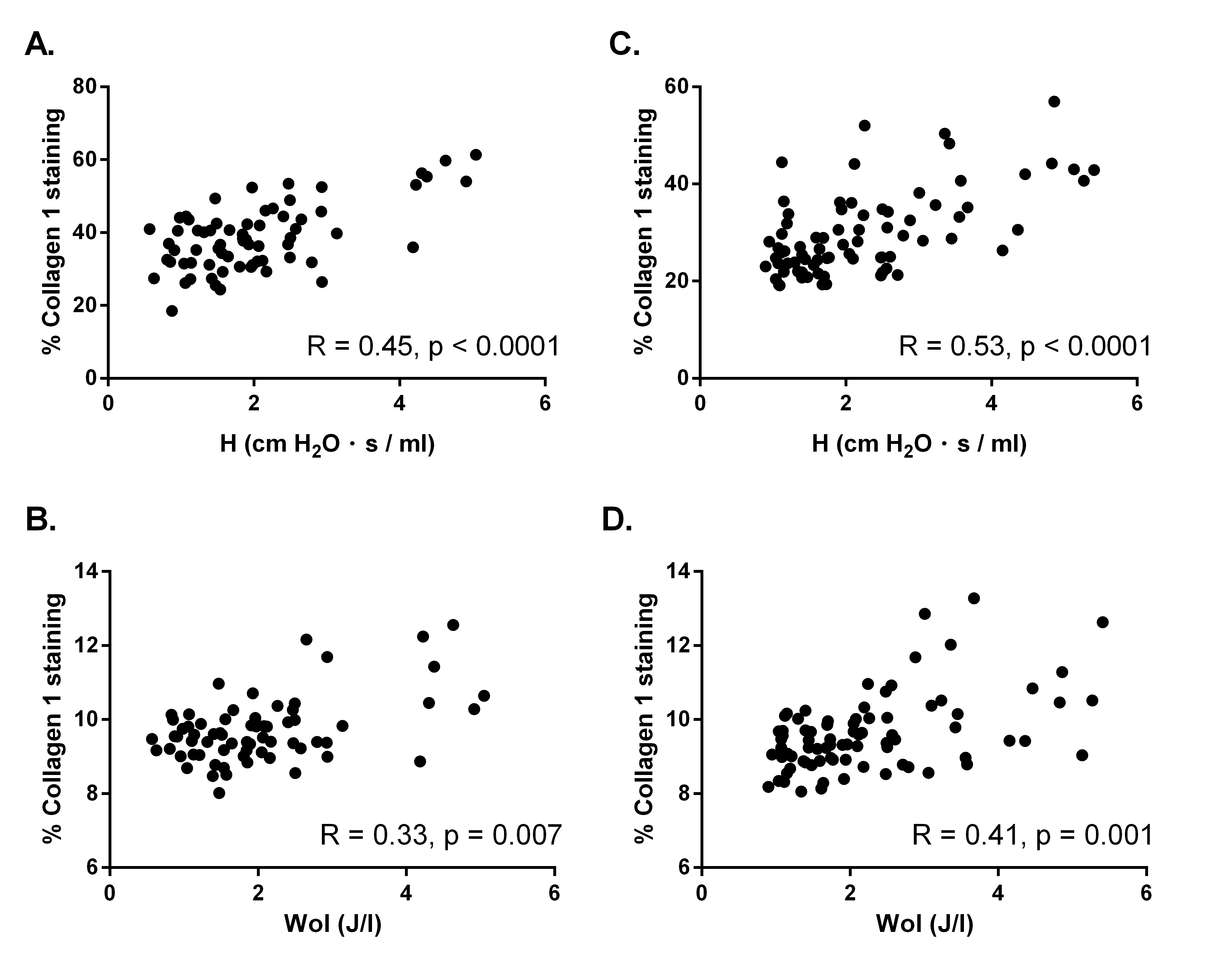

Supplement: Figure S4 — Changes in lung mechanics correlate to changes in lung fibrosis. Changes in H (A) and volume normalized work of inflation (WoI) (B) after a single bleomycin instillation significantly correlated to the amount of lung tissue stained positive for collagen I as determined by Pearson correlation coefficients (R). The correlations for H (C) and WoI (D) with collagen I were similar after repetitive bleomycin instillations. (TIF) [file pone.0059348.s004.tif]

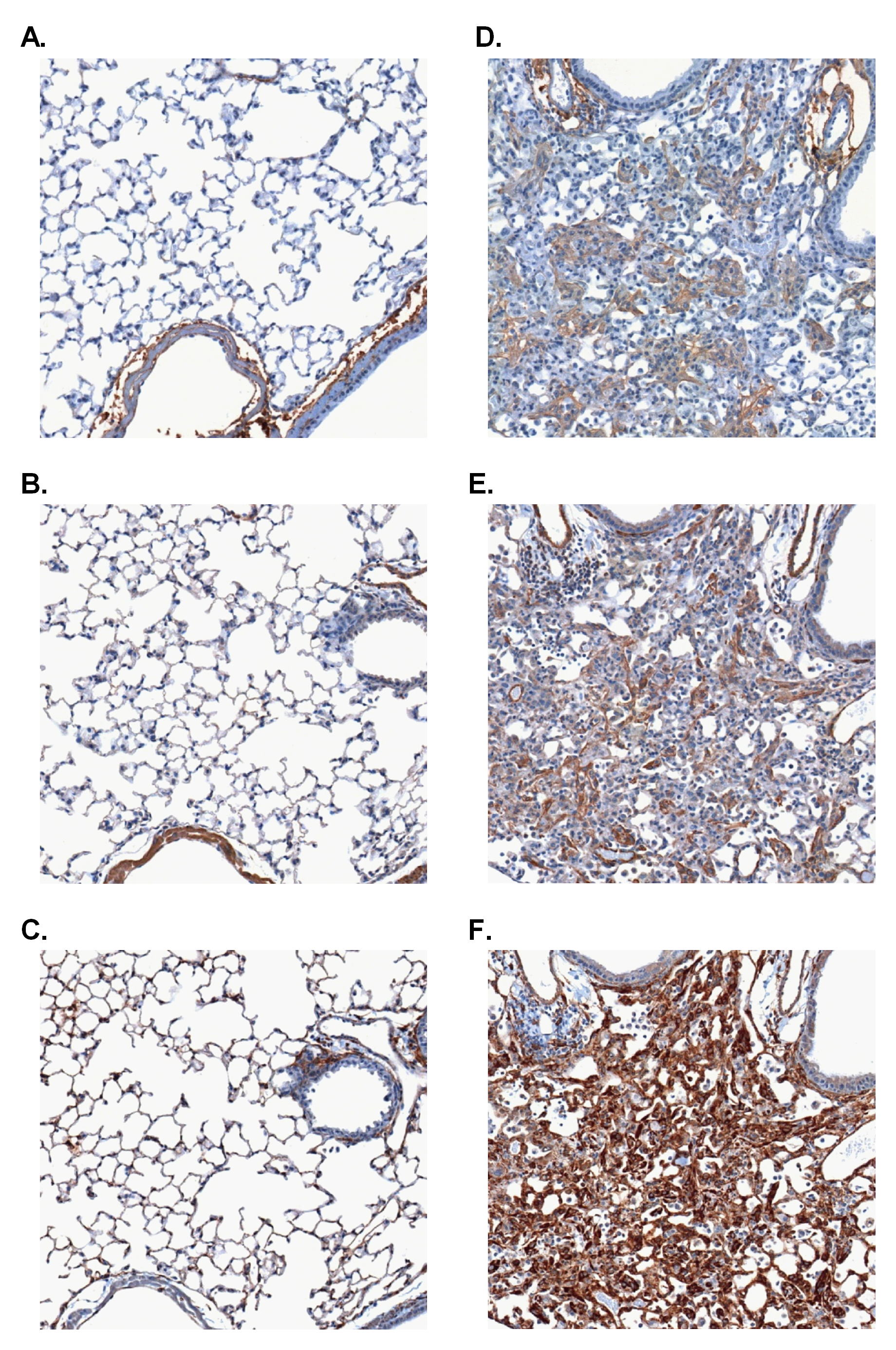

Supplement: Figure S5 — Bleomycin increased collagen deposition and myofibroblast activation. Serial lung tissue sections from one representative animal illustrate that areas positive for collagen I (D) were also positive for α-smooth muscle actin (E), as well as HSP47 (F). As a reference, sections from saline treated animals stained for collagen I (A), α-smooth muscle actin (B), and HSP47 (C) are also shown. Images were captured at 200X magnification. (TIF) [file pone.0059348.s005.tif]

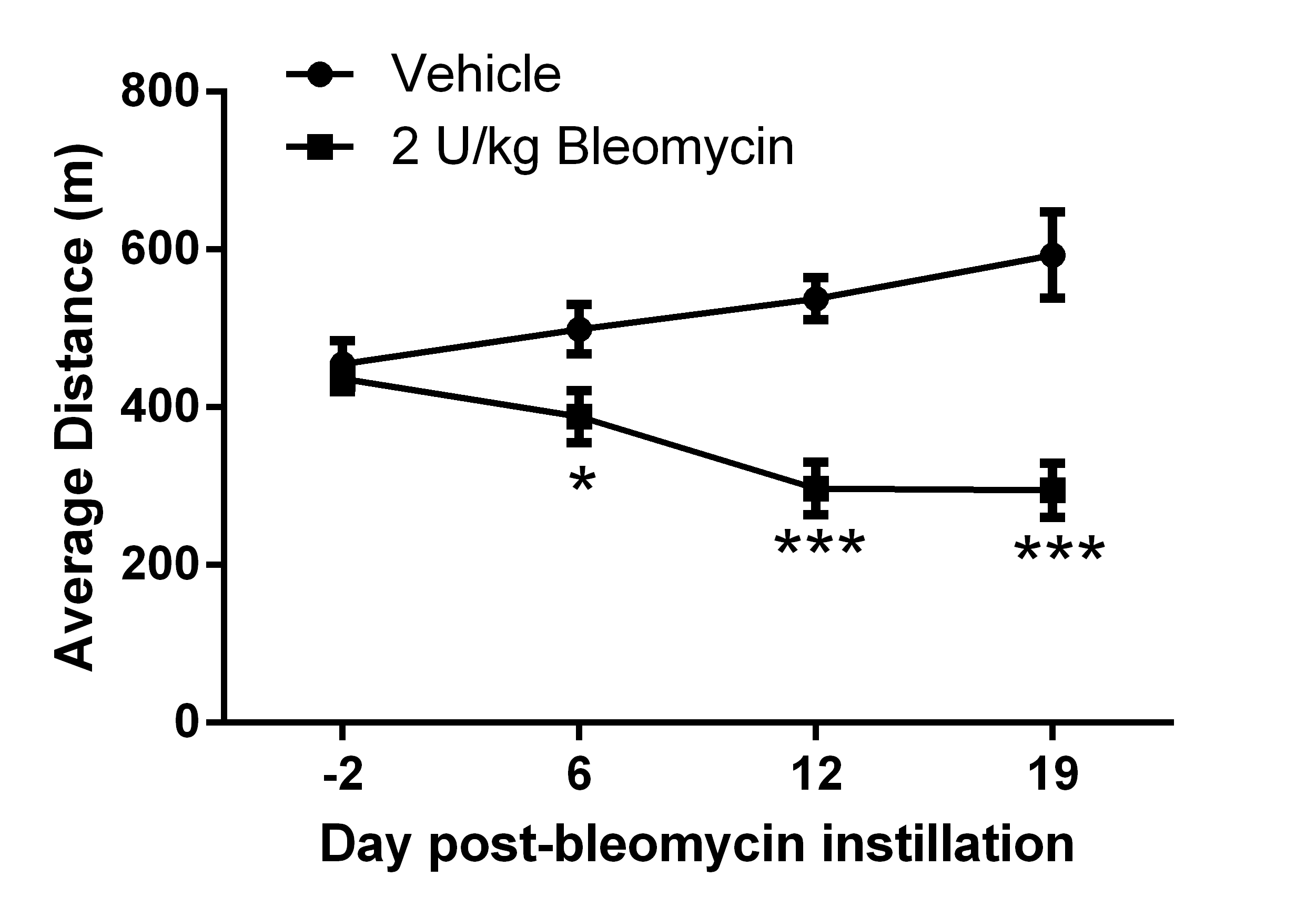

Supplement: Figure S6 — Exercise capacity is reduced in bleomycin treated mice. The exercise capacity of mice treated with saline (filled circles) or 2 U/kg bleomycin (filled squares) was measured on a motorized treadmill. Data expressed as mean ± SEM of n = 19–20. Significance relative to the saline treated was determined using a one-way ANOVA and Dunnett’s post-hoc test and is denoted as, *p<0.05; or ***, p<0.001. (TIF) [file pone.0059348.s006.tif]

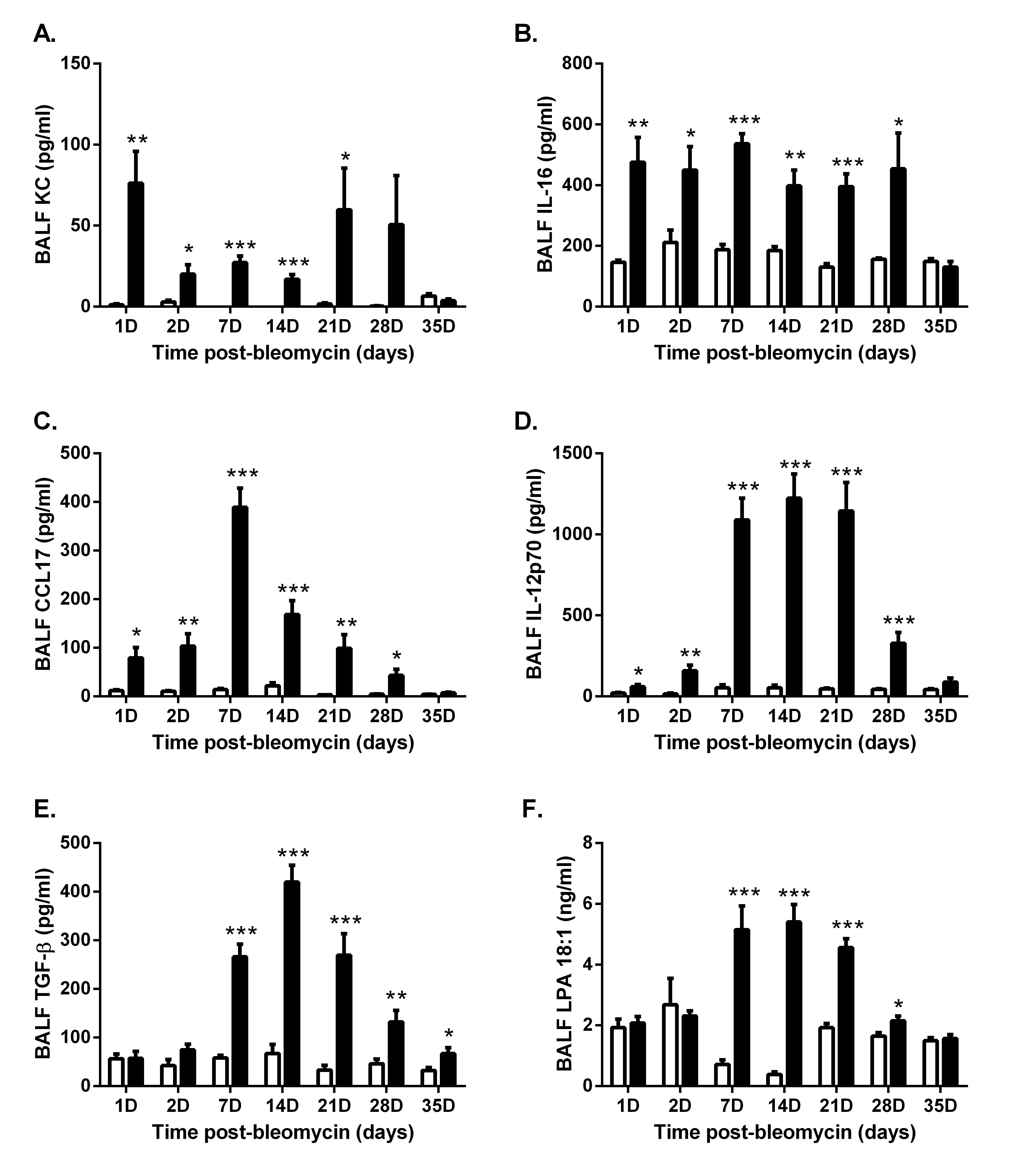

Supplement: Figure S7 — Bleomycin induces cytokine, chemokine, and pro-fibrotic mediator secretion in the BALF. BALF levels of KC (A), IL-16 (B), CCL17 (TARC) (C), total IL-12 (D), TGFβ (E), and LPA (18:1 isoform) (F)were measured. Saline treated controls represented by white bars and bleomycin treated animals by black bars. The following cytokines had no measurable change: IFN-g, TNF-α, IL-1β, IL-2, IL-4, IL-5, IL-10, GM-CSF, VEGF, IL-13, IL-17 (data not shown). Data are expressed as mean ± SEM of n = 8 mice. Significance (relative to the time-matched control at each time point) was determined using a Student’s t-test and is denoted as, *p<0.05. (TIF) [file pone.0059348.s007.tif]

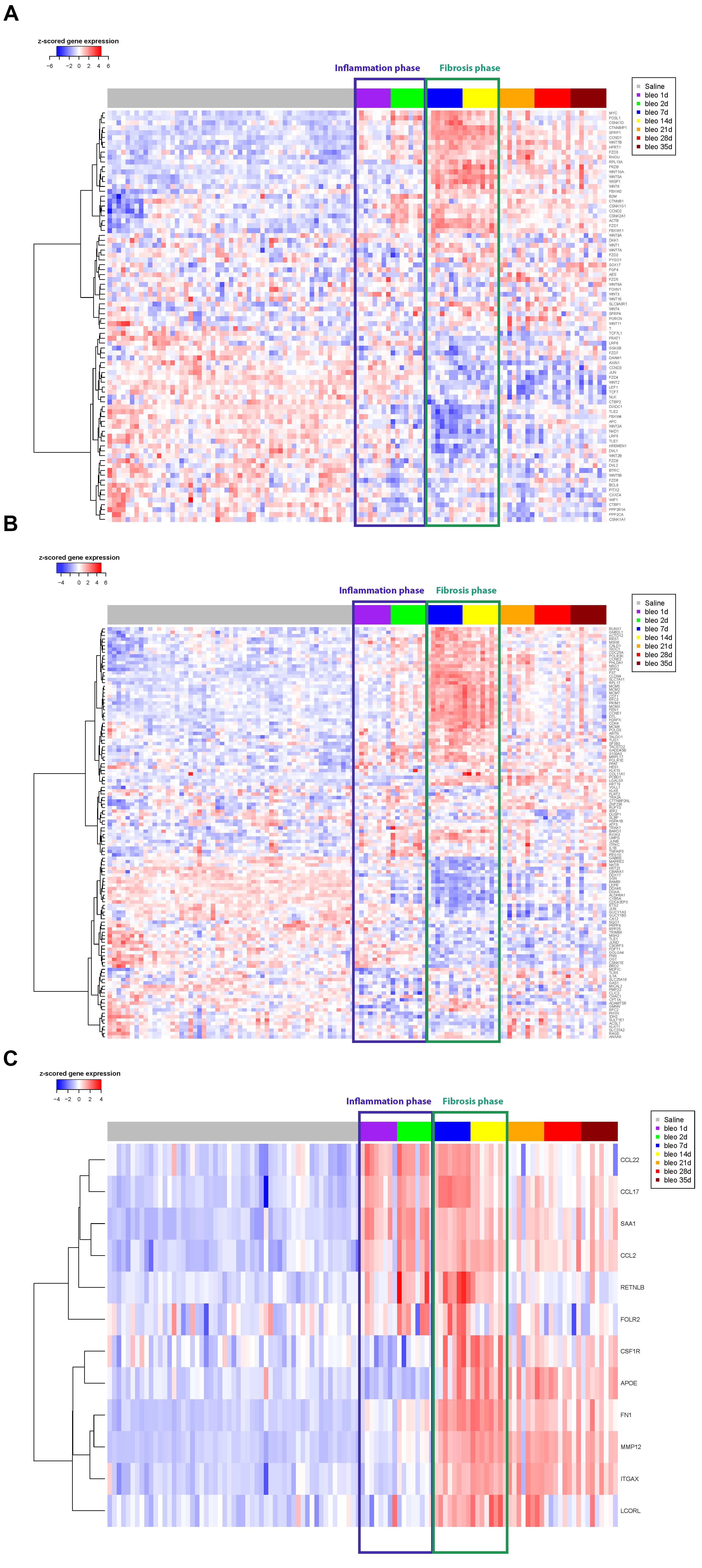

Supplement: Figure S8 — Supervised hierarchical clustering of custom panels of genes across all mouse samples. Clustering of genes was performed for a panel of (A) genes involved in Wnt signaling, (B) genes altered downstream of PI3 kinase, and (C) genes involved in alternative macrophage activation. Samples are ordered based on treatment and time point. (TIF) [file pone.0059348.s008.tif]

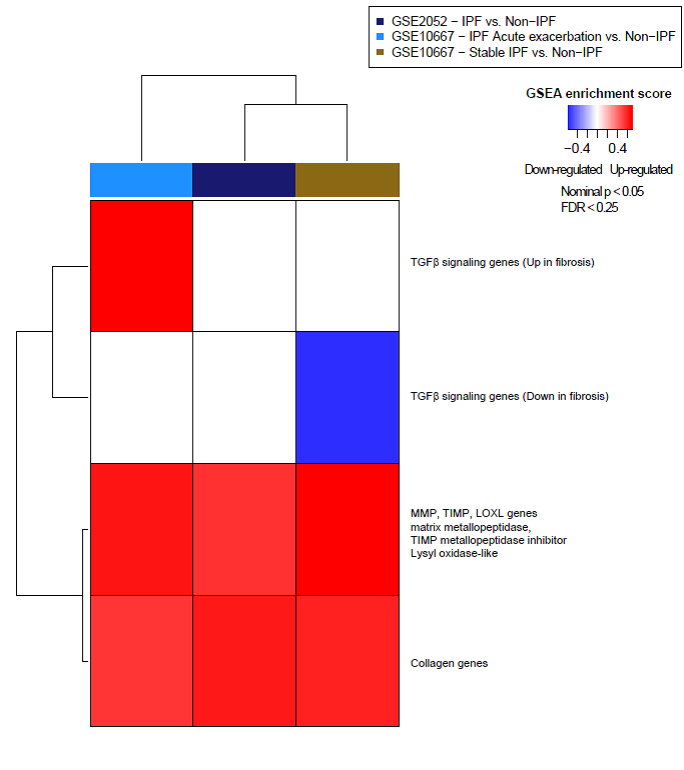

Supplement: Figure S9 — Heatmap of gene set enrichment of custom signatures in clinical IPF datasets. GSEA was performed for gene sets of custom gene panels from fibrosis-related mechanisms including MMP, LOXL, collagen, and TGFβ signaling. Enrichment of each gene set (denoted in rows) was determined against ranked lists of genes from clinical datasets comparing IPF vs. non-IPF conditions from two datasets (GSE2052, GSE10667, denoted in columns). Enrichment scores were plotted in a heatmap where gene sets enriched in IPF samples (nominal p<0.05, FDR <0.25) were denoted in red while gene sets enriched in non-IPF samples were denoted in blue. Intensity of each cell was based on enrichment score (calculated in GSEA). (TIF) [file pone.0059348.s009.tif]

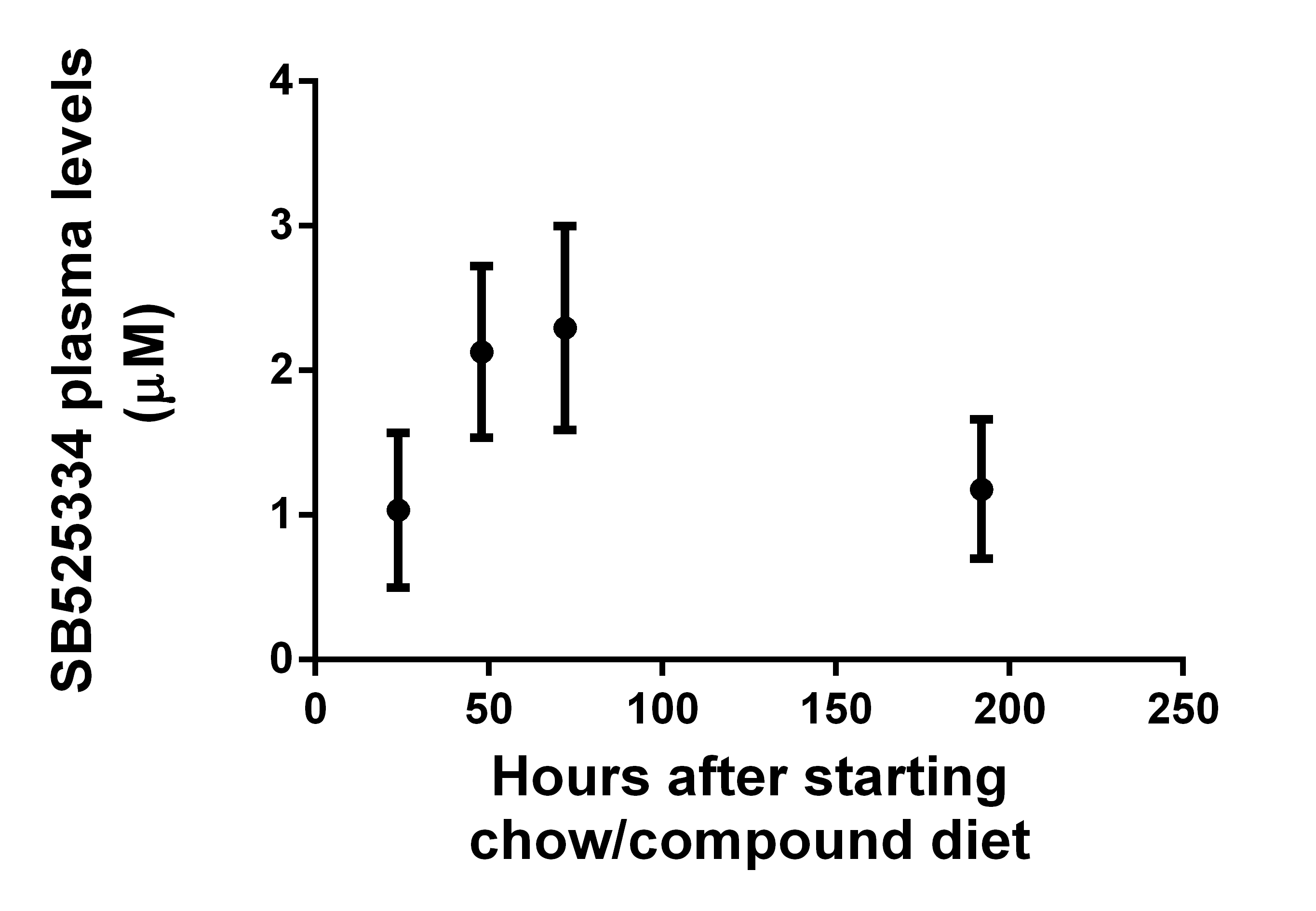

Supplement: Figure S10 — Plasma exposure levels for SB525334 in bleomycin treated mice after initiating a diet using Purina Rodent Chow #5001 mixed with SB525334. (TIF) [file pone.0059348.s010.tif]
