# Supplementary material for: Bleomycin Induces Molecular Changes Directly Relevant to Idiopathic Pulmonary Fibrosis: A Model for “Active” Disease
Source: PLoS One. 2013 Apr 2;8(4):e59348. doi: 10.1371/journal.pone.0059348 (PMC3614979; doi:10.1371/journal.pone.0059348)
Supplement: Data File S1 — A union of leading edge genes from GSEA of bleomycin-induced gene sets in IPF vs. non-IPF subject comparisons from two clinical cohorts (GSE2052, GSE10667). This alphabetized list of genes corresponds to a union of the genes highlighted in Figure 6. (PDF) [file pone.0059348.s011.pdf]

Data File S1. A union of leading edge genes from GSEA of bleomycin-induced gene sets in IPF vs. non-IPF subject comparisons from two clinical cohorts (GSE2052, GSE10667). This alphabetized list of genes corresponds to a union of the genes highlighted in Figure 6.

AADAC	C3AR1	CENPN	CTSK	FN1	KIF20A	NCAPG	SAA1	TFEC
ADAM12	CAPN6	CENPP	CXCL12	FOXM1	KIF22	NCAPG2	SCAMP5	THBS1
ADAMTS12	CASC5	CEP55	CXCL13	FST	KIF23	NEIL3	SERPINA10	THBS2
ADAMTS4	CCDC80	CFI	CYP7B1	GAS2L3	KIF2C	NEK6	SFRP1	THBS4
ANKRD34B	CCDC99	CGREF1	DARC	GDF15	KLHDC8A	NNAT	SGOL1	TIMP1
ANLN	CCNA2	CH25H	DBN1	GINS2	KNTC1	NUSAP1	SH3PXD2B	TK1
ASF1B	CCNB2	CHEK1	DCLK1	GJB3	KYNU	P4HA3	SHC4	TNC
ASPM	CCNE1	CHL1	DDIT4L	GJB5	LAIR1	PAPPA2	SHCBP1	TNFAIP8L1
ATG9B	CCNF	CHSY3	DEPDC1	GPNMB	LAMA1	PBK	SIGLEC1	TNFSF11
ATP6V0D2	CCNG1	CILP	DLGAP5	GREM1	LHFPL2	PDGFC	SLAMF9	TPX2
AURKA	CCR5	CKAP2L	DSCC1	GTSE1	LRRC15	PHEX	SLC26A4	TREM2
AURKB	CD276	CLEC5A	E2F7	HMMR	LUM	PI15	SLC37A2	TRIP13
BCAT1	CDC20	CLSPN	E2F8	IBSP	MAFB	PIF1	SLC39A14	TTK
BHLHE22	CDC25C	COL15A1	ELN	IGF1	MARK1	PKP1	SLC7A11	TUB
BUB1	CDCA2	COL1A2	ESCO2	IGJ	MASTL	PLCD4	SLFN13	TUBB3
C12orf48	CDCA3	COL24A1	ESPL1	IL12B	MEGF10	PMCH	SMC2	UBD
C12orf53	CDCA5	COL3A1	EXO1	IL1RN	MELK	PROX1	SPAG5	UBE2C
C12orf75	CDCA8	COL5A1	F7	IL2RA	MEX3A	PRRX2	SPC24	UBE2T
C15orf48	CDK1	COL5A2	FAM3B	ITGAX	MMP10	PRUNE2	SPC25	UHRF1
C1orf135	CDKN3	COL8A1	FBN1	ITGB6	MMP13	PSRC1	SPINK1	VCAN
C1QB	CENPE	CPS1	FBN2	KIAA0101	MMP14	PTGER3	SPP1	VSIG4
C1QC	CENPF	CPXM1	FCGR2A	KIAA1199	MMP2	RAD51	STAC2	WISP1
C1QTNF3	CENPH	CRABP1	FCGR2B	KIAA1524	MTHFD2	RAD51AP1	STIL	ZRANB3
C1QTNF6	CENPK	CRH	FIGNL1	KIF11	MYO5A	RGS1	SYT12	ZWILCH
C2orf40	CENPM	CTHRC1	FMOD	KIF18B	NAV3	RHBDL2	TFAP2A	
