# Supplementary material for: Bleomycin Induces Molecular Changes Directly Relevant to Idiopathic Pulmonary Fibrosis: A Model for “Active” Disease
Source: PLoS One. 2013 Apr 2;8(4):e59348. doi: 10.1371/journal.pone.0059348 (PMC3614979; doi:10.1371/journal.pone.0059348)
Supplement: Data File S2 — Canonical pathways enriched among the leading edge genes from GSEA of bleomycin-induced gene sets in IPF vs. non-IPF subject comparisons from two clinical cohorts (GSE2052, GSE10667). The canonical pathways, p value of the enrichment of leading edge genes in these pathways, and the leading edge genes contained within the pathways are highlighted in this table. (PDF) [file pone.0059348.s012.pdf]

Data File S2. Canonical pathways enriched among the leading edge genes from GSEA of bleomycin-induced gene sets in IPF vs. non-IPF subject comparisons from two clinical cohorts (GSE2052, GSE10667). The canonical pathways, p value of the enrichment of leading edge genes in these pathways, and the leading edge genes contained within the pathways are highlighted in this table.

Canonical Pathway	P value	Leading edge genes within the pathway
16_M_Phase_- REACTOME	1.23E-21	AURKB,KIF2C,KIF20A,KNTC1,CENPF,CENPE,KIF23,CDC20,BUB1,CDCA8,SPC24,CENPM,SPC25,CENPP,SGOL1,CENPN,CCDC99,CASC5,ZWILCH,CENPH,CENPK
16_Mitotic_Prometaphase_- REACTOME	3.55E-19	AURKB,KIF2C,KNTC1,CENPF,CENPE,CDC20,BUB1,CDCA8,SPC24,CENPM,SPC25,CENPP,SGOL1,CENPN,CCDC99,CASC5,ZWILCH,CENPH,CENPK
16_Cell_Cycle__Mitotic_- REACTOME	2.08E-18	CCNA2,AURKB,UBE2C,KIF2C,CDK1,KIF20A,KNTC1,CENPF,CENPE,KIF23,CDC20,CDC25C,CCNB2,BUB1,CDCA8,SPC24,CENPM,SPC25,AURKA,GINS2,CCNE1,CENPP,SGOL1,CENPN,CCDC99,CASC5,ZWILCH,CENPH,CENPK
16_DNA_Replication_- REACTOME	5.94E-17	CCNA2,AURKB,KIF2C,KIF20A,KNTC1,CENPF,CENPE,KIF23,CDC20,BUB1,CDCA8,SPC24,CENPM,SPC25,GINS2,CENPP,SGOL1,CENPN,CCDC99,CASC5,ZWILCH,CENPH,CENPK
16_Mitotic_M_M_G1_phases_- REACTOME	8.30E-16	AURKB,KIF2C,KIF20A,KNTC1,CENPF,CENPE,KIF23,CDC20,BUB1,CDCA8,SPC24,CENPM,SPC25,CENPP,SGOL1,CENPN,CCDC99,CASC5,ZWILCH,CENPH,CENPK
223_Aurora_B_signaling_- NCI_NATURE	9.13E-12	AURKB,KIF2C,KIF20A,NCAPG,KIF23,BUB1,CDCA8,AURKA,SGOL1,SMC2
18_Polo_like_kinase_signaling_events_in_the_cell_cycle_- NCI_NATURE	1.21E-11	AURKB,CDK1,DLGAP5,KIF20A,CENPE,TPX2,CDC20,CDC25C,BUB1,SPC24,CLSPN,AURKA,CCNE1,SGOL1
18_PLK1_signaling_events_- NCI_NATURE	9.27E-11	AURKB,CDK1,DLGAP5,KIF20A,CENPE,TPX2,CDC20,CDC25C,BUB1,SPC24,CLSPN,AURKA,SGOL1
18_Signaling_by_Aurora_kinases_- NCI_NATURE	7.00E-10	AURKB,KIF2C,DLGAP5,KIF20A,NCAPG,TPX2,KIF23,BUB1,CDCA8,AURKA,SGOL1,SMC2
75_Integrin_cell_surface_interactions_- REACTOME	3.40E-08	TNC,FBN1,ITGB6,SPP1,COL1A2,LAMA1,IBSP,THBS1,ITGAX,FN1
157_Signaling_by_PDGF_- REACTOME	3.81E-08	COL5A2,THBS2,PDGFC,SPP1,COL3A1,COL5A1,COL1A2,THBS1,THBS4
414_Syndecan_4_mediated_signaling_events_- NCI_NATURE	8.75E-07	TNC,CXCL12,ADAM12,LAMA1,THBS1,FN1
277_FOXM1_transcription_factor_network_- NCI_NATURE	1.91E-06	AURKB,CDK1,CENPF,CCNB2,FOXM1,MMP2
154_Chk1_Chk2_Cds1__mediated_inactivation_of_Cyclin_B_Cdk1_complex_- REACTOME	5.50E-06	CHEK1,CDK1,CDC25C
318_Kinesins_- REACTOME	9.15E-06	KIF2C,KIF20A,KIF11,KIF23,KIF22
4_Cell_Cycle_Checkpoints_- REACTOME	4.92E-05	UBE2C,CHEK1,CDK1,CDC20,CDC25C,CCNB2,CLSPN,CCNE1
154_G2_M_DNA_damage_checkpoint_- REACTOME	7.45E-05	CHEK1,CDK1,CDC25C
51_G2_M_Checkpoints_- REACTOME	0.000102285	CHEK1,CDK1,CDC25C,CCNB2,CLSPN
85_Deposition_of_New_CENPA_containing_Nucleosomes_at_the_Centromere_- REACTOME | Nucleosome_assembly - REACTOME	0.00012823	CENPP,CENPN,CASC5,CENPH,CENPK
4_APC_C_mediated_degradation_of_cell_cycle_proteins_- REACTOME | Regulation_of_mitotic_cell_cycle - REACTOME	0.000297569	CCNA2,AURKB,UBE2C,CDK1,CDC20,AURKA
154_G2_M_DNA_replication_checkpoint_- REACTOME	0.000371886	CDK1,CCNB2
16_Mitotic_Telophase__Cytokinesis_- REACTOME	0.000371886	KIF20A,KIF23
154_Cyclin_A_B1_associated_events_during_G2_M_transition_- REACTOME	0.0004606	CDK1,CDC25C,CCNB2
1_Proteogylcan_syndecan_mediated_signaling_events_- NCI_NATURE	0.000602167	IGF1,TNC,CCNA2,CXCL12,VCAN,IL2RA,SPP1,MMP14,ADAM12,GDF15,MMP2,COL1A2,LAMA1,CCNE1,CCNG1,THBS1,FCGR2B,FN1
266_E2F_transcription_factor_network_- NCI_NATURE	0.000649008	CCNA2,CDK1,TK1,E2F7,CCNE1
377_Regulation_of_Insulin_like_Growth_Factor__IGF__Activity_by_Insulin_like_Growth_Factor_Binding_Proteins__IGFBPs__- REACTOME	0.000696987	IGF1,MMP2,PAPPA2
255_Cyclin_B2_mediated_events_- REACTOME	0.000738272	CDC25C,CCNB2
30_Hemostasis_- REACTOME	0.000814646	IGF1,KIF2C,KIF20A,F7,KIF11,CENPE,KIF23,KIF22,TIMP1,COL1A2,SLC7A11,THBS1,ITGAX,FN1
124_NCAM_signaling_for_neurite_out_growth_- REACTOME	0.00105806	CDK1,COL5A2,COL3A1,COL5A1,COL1A2
360_Phosphorylation_of_Emi1_- REACTOME	0.001221364	CDK1,CDC20
21_APC_C_Cdc20_mediated_degradation_of_Cyclin_B_- REACTOME	0.001372755	UBE2C,CDK1,CDC20
124_NCAM1_interactions_- REACTOME	0.001342411	COL5A2,COL3A1,COL5A1,COL1A2
